# Intrathyroidal feedforward and feedback network regulating thyroid hormone synthesis and secretion

**DOI:** 10.3389/fendo.2022.992883

**Published:** 2022-09-15

**Authors:** Li Jing, Qiang Zhang

**Affiliations:** ^1^ Department of Toxicology and Hygienic Chemistry, School of Public Health, Capital Medical University, Beijing, China; ^2^ Gangarosa Department of Environmental Health, Rollins School of Public Health, Emory University, Atlanta, GA, United States

**Keywords:** thyroid hormone, feedforward, feedback, thyrocyte, intrathyroidal regulation

## Abstract

Thyroid hormones (THs), including T4 and T3, are produced and released by the thyroid gland under the stimulation of thyroid-stimulating hormone (TSH). The homeostasis of THs is regulated *via* the coordination of the hypothalamic-pituitary-thyroid axis, plasma binding proteins, and local metabolism in tissues. TH synthesis and secretion in the thyrocytes-containing thyroid follicles are exquisitely regulated by an elaborate molecular network comprising enzymes, transporters, signal transduction machineries, and transcription factors. In this article, we synthesized the relevant literature, organized and dissected the complex intrathyroidal regulatory network into structures amenable to functional interpretation and systems-level modeling. Multiple intertwined feedforward and feedback motifs were identified and described, centering around the transcriptional and posttranslational regulations involved in TH synthesis and secretion, including those underpinning the Wolff-Chaikoff and Plummer effects and thyroglobulin-mediated feedback regulation. A more thorough characterization of the intrathyroidal network from a systems biology perspective, including its topology, constituent network motifs, and nonlinear quantitative properties, can help us to better understand and predict the thyroidal dynamics in response to physiological signals, therapeutic interventions, and environmental disruptions.

## Introduction - homeostatic regulation of thyroid hormones

Thyroid hormones (THs), including thyroxine (T4) and 3,3′,5-triiodo-L-thyronine (T3), are produced and released by thyrocytes-containing follicles in the thyroid gland. T4 and T3 are transported through the blood circulation to target tissues, where T4 is converted intracellularly to T3, the active hormone. In the nucleus, T3 binds to TH receptors (TR) that are dimerized with retinoid X receptors (RXR) on the TH response elements (TREs) of a myriad of target genes to regulate their transcription. THs are essential for normal development, growth, and metabolism of almost all tissues. They regulate cardiovascular and energetic homeostasis (1, 2), female reproduction (3), and the development of the nervous and skeletal systems (4, 5). Insufficient or excessive levels of THs result in a multitude of developmental and metabolic diseases. Therefore, robust control of TH homeostasis is essential.

TH homeostasis is maintained both globally and locally through multiple mechanisms that coordinately regulate the production, secretion, distribution, and metabolism of THs. These mechanisms include systemic feedback regulation of TH synthesis and secretion by the thyroid-stimulating hormone (TSH) through the hypothalamic-pituitary-thyroid (HPT) axis, buffering by the TH binding proteins (THBPs) in the blood circulation, and local feedback regulation by metabolic enzymes of THs in peripheral tissues (6–9). Homeostatic compensation through the HPT feedback can take days or even several weeks to complete given the long half-lives of T3 and T4 in humans (10–12). In contrast, the fast binding between THs and THBPs renders the adjustment through buffering almost instantaneous, in seconds to minutes (13, 14). These multi-layered, cross-scale regulations, operating on different time scale and in different anatomical locations, coordinate with one another forming the basic regulatory framework of TH homeostasis.

### HPT feedback regulation

The classical feedback regulation of the HPT axis is the primary mechanism for chronically maintaining the circulating free TH levels within the physiological range (15). The TSH-releasing hormone (TRH) is synthesized as a neurohormone in the paraventricular nucleus (PVN) of the hypothalamus. Released in the median eminence and transported *via* the hypophyseal portal vasculature, TRH reaches the anterior pituitary and stimulates the thyrotropes to secrete TSH into the systemic blood circulation. In turn, TSH stimulates the synthesis and secretion of T4 and T3 in the thyroid gland. Secreted THs then reach the brain where they inhibit the production and release of both TRH and TSH (16). For instance, in the TRH neurons located in the hypothalamic paraventricular nucleus, T3 represses the transcription of the TRH gene *via* TH receptor β2 (TRβ2) (17). In the pituitary thyrotrophs, T3 represses the transcription of the TSHβ subunit by inhibiting *via* TRβ2 the autoregulation of GATA2, a key transcription factor that mediates the TRH-stimulated TSH production (18). Additionally, in the tanycytes located in the median eminence, T3 can upregulate the expression of pyroglutamyl peptidase II, which is a highly specific TRH peptidase that degrades the locally released TRH before it reaches the portal vein system (19). In summary, T3-mediated inhibition of TRH and TSH completes the negative feedback loop of the HPT axis, which can maintain the steady-state circulating free TH levels within a very narrow 2-3 fold range in humans (20, 21).

### Buffering by TH binding proteins

Acute or transient perturbation of circulating free THs is resisted by the THBPs. In the human blood, only a small fraction of THs exists in the free, unbound form, while nearly 99.97% of T4 and 99.70% of T3 are bound to specific THBPs (22), including thyroxine-binding globulin (TBG), transthyretin (TTR), and albumin (23). TBG is the most abundant and has the greatest affinity for THs, followed by TTR and albumin (24). Overall, TBG could account for 75%, TTR 15%, and albumin 10% of total THs in the plasma, with a negligible amount distributed in lipoproteins (25). The THBPs can shield THs from the aqueous environment in the plasma. More importantly, just like a pH buffer system, the THBPs serve as a reservoir to buffer free T4 and T3 concentrations against transient fluctuations and ensure steady delivery of THs through the target tissues (26, 27). The buffering operates in a time scale of seconds to minutes, which is much faster than the feedback action of the HPT axis. However, since the long-term free TH levels are determined by the HPT axis, abnormality in the abundances or binding affinities of THBPs only result in changes in the concentrations of total, not free, THs in the blood, and the patients are in most cases clinically euthyroid (28).

### Local feedback regulation of TH metabolism

Despite that free THs in the circulation are robustly regulated by the HPT feedback and THBPs, it is T4 and particularly T3 in the cells that ultimately drive the biological outcomes. THs in the circulation are taken up by cell membrane transporters and metabolized intracellularly by deiodinases (DIO) which can either activate or deactivate THs (8, 29). The intracellular TH concentrations in peripheral tissues are controlled by multiple local feedbacks regulating the activities and expression levels of DIOs. T3 can repress the gene transcription of DIO2, the primary activating enzyme that converts T4 into T3, therefore forming a negative feedback loop to keep intracellular T3 within a range (30–32). DIO2 transcription is also inhibited by rT3 (33). In addition, T3 can induce DIO1 transcription by binding to two TREs in the gene promoter in tissues including the liver (34–36). This induction may form a positive feedback *via* DIO1-mediated production of T3 from T4, thus exacerbating hyperthyroid conditions (32). However, because DIO1 can also deactivate T4 into rT3 and deactivate T3 into T2, a local negative feedback control cannot be ruled out. T4 and rT3 also inhibit DIO2 by promoting its ubiquitination, which inhibits DIO2 activity and targets it for proteasomal degradation (37–40), forming an incoherent feedforward motif with T3 as the output. Lastly, it has been reported that an increase in serum T3 could upregulate DIO3 expression in the brain, increasing local T3 clearance, forming another local feedback control of T3 (41–43). In addition to deiodination, in hepatocytes feedback regulation of THs also appears to be mediated by glucuronidation *via* inducible UDP-glucuronosyltransferase (UGTs) (44–47). In summary, THs can regulate the activities and abundances of DIOs and certain UGTs in peripheral tissues in a feedback or feedforward manner to maintain their local levels.

The three global and local mechanisms described above are crucial to maintaining THs at appropriate physiological levels in the circulation and target tissues. For the HPT axis to sustain circulating free THs within the normal range, the regulation of TH synthesis and secretion by TSH in the thyroid gland plays a central role. This process occurs in thyroid follicles, involving iodide uptake, iodination of thyroglobulin (TG), proteolysis of TG into T3 and T4, and their release into the systemic circulation. The process is exquisitely regulated through an elaborate molecular network of enzymes, transporters, signal transduction machineries, and transcription factors (TFs), which form multiple intertwined feedback and feedforward structures. Feedback and feedforward are key network motifs that underpin a variety of dynamic behaviors, including signal amplification, multistability, adaptation, homeostasis, oscillation, fold-change detection, signaling delay and acceleration, threshold and nonmonotonic effect etc. (48, 49). Multiple motifs coupled together as seen in complex networks can generate even richer dynamics and advanced functions. Understanding the systems biology, including the organization and quantitative properties, of the complex intrathyroidal network will help ascertain not only the thyroidal responses to physiological signals such as TSH, but also the potential adverse health outcomes and risks of environmental endocrine disrupting chemicals (EDCs) that directly perturb the thyroid gland. In this article, we intended to review and synthesize the relevant literature, and organize the intrathyroidal regulatory network of TH synthesis and secretion into structures that are amenable not only to functional interpretation but also to dynamical modeling in future. We started the review by first introducing the TH synthesis and secretion pathway, followed by describing the TSH-stimulated signal transduction components and TFs that regulate the expression and activities of the enzymes and proteins involved in TH synthesis and secretion. The multiple transcriptional and posttranslational feedforward and feedback circuits between TFs and enzymes were then depicted, including the feedforward and feedback regulations underpinning the Wolff-Chaikoff and Plummer effects of high iodine and TG-mediated inhibition. Lastly, perspectives were provided on how quantitative characterization of the intrathyroidal network can help us to better understand TH regulation, thyroid disease, and adverse effects of thyroid EDCs. It should be noted though that many of the regulations reviewed here are based on *in vitro* and *in vivo* studies in a variety of species, particularly rat thyroid cell lines, thus caution should be exercised when generalization is made. This review is unique in that it is not intended to provide a comprehensive coverage and enumeration of the molecular biology details of known regulations in thyrocytes, but to focus on the systems-level structures, potential functions, and biological significances that these regulations as a whole can furnish when connected into complex networks.

## The TH synthesis and secretion pathway

### Iodide uptake, exportation, and organification

Thyroid follicles are the functional units of the thyroid gland. They are spherical structures comprising a monolayer of epithelial cells (thyrocytes) that enclose an extracellular lumen filled with TG colloid (50). Each follicle is encapsulated by a rich network of capillaries. Uptake of circulating I^-^ by thyroid follicles is mediated by the Na^+^/I^-^ symporter (NIS), a glycoprotein located on the basolateral plasma membrane of the thyrocytes and encoded by the solute carrier family 5 member 5 (*SLC5A5*) gene (51). The active uptake of I^-^ by NIS is Na^+^-dependent, which couples the energy released by the inward translocation of Na^+^ down its chemical gradient to the simultaneous inward translocation of I^-^ against its electrochemical gradient (52). Inside the thyrocytes, I^-^ diffuses to the apical side of the cell, where it is taken up and transported into the follicular lumen by transporters located on the apical plasma membrane, including pendrin (PDS), anoctamin-1 (ANO1), and SLC26A7 (53, 54). PDS is an anion transporter encoded by the *SLC26A4* gene (55, 56). ANO1 is a calcium-activated chloride channel, which has been demonstrated to mediate I^-^ efflux across the apical membrane of thyrocytes (54). SLC26A7, a member transporter belonging to the same family as SLC26A4, is an anion exchanger with affinity for I^-^ and chloride (56).

Once in the follicular lumen, I^-^ is organified by key enzymatic complexes anchored on the apical surface of the thyrocytes, containing dual oxidase (DUOX) and thyroid peroxidase (TPO). A member of the NADPH oxidase (NOX) family, DUOX1 and DUOX2 utilize the protons provided by intracellular NADPH to generate and release H_2_O_2_ into the lumen. The maturation and function of DUOX1 and DUOX2 require DUOX maturation factor 1 (DUOXA1) and DUOXA2, respectively, which are chaperone proteins. By forming enzymatic complexes DUOX1/DUOXA1 and DUOX2/DUOXA2, the chaperones promote the correct maturation of DUOX1 and DUOX2 for their endoplasmic reticulum (ER) exit to the cell surface (57). Although similar in structure, the DUOX1 and DUOX2 proteins are encoded by two separate genes with fairly different regulatory mechanisms (58). Compared to DUOX1/DUOXA1, DUOX2/DUOXA2 has higher enzymatic activities and is the predominant form in the thyroid (57, 59). TPO utilizes H_2_O_2_ generated by DUOX/DUOXA to catalyze the oxidation of I^-^ and subsequent iodination of the tyrosine residues of soluble TG molecules nearby (60). The cooperation between DUOX/DUOXA and TPO is facilitated *via* the formation of thyroxisome, a complex comprising DUOX/DUOXA, TPO, and caveolin-1 (CAV1). CAV1 is required for the correct positioning of TPO and DUOX on the apical membrane (61, 62), bringing the two together in the thyroxisome (63). The close proximity between DUOX and TPO makes newly produced H_2_O_2_ readily captured by TPO and reduces potential oxidative damage by H_2_O_2_ that would otherwise diffuse afar (64, 65). At the end of the organification process, I^-^ is incorporated to specific tyrosine residues in the TG molecule, forming intramolecular mono-iodotyrosine (MIT) and di-iodotyrosine (DIT). TPO then further catalyzes the coupling of MIT and DIT in the same TG molecule to produce intramolecular T3 and T4 (66).

### TG dynamics in the lumen

As the macromolecular precursor of THs, TG is synthesized and processed in ER to form dimers with N-linked glycoside, followed by further processing in the Golgi apparatus where modifications of the carbohydrate moieties occur (67). Mature TG molecules are transported *via* vesicles from the trans-Golgi network to the apical surface of the thyrocytes, then they are released into the lumen and stored there as the predominant protein content of the colloid (67). Transportation of TG to the lumen may also be mediated by asialoglycoprotein receptor (ASGPR) (68).

#### TG structure

TG is a noncovalent homodimer (660 kDa) with a high degree of glycosylation. Each TG monomer contains 67 tyrosine residues, of which ~30 can be iodinated, among which four are hormonogenic acceptors and five are donors. In human TG, the four acceptor tyrosine residues are localized at positions 24, 1310, 2573, and 2766 (69, 70), and the donor tyrosine residues at 108, 149, 234, 2540, and 2766 (71, 72). After iodination, the aromatic ring of a donor DIT or MIT is transferred by TPO to a proximal acceptor DIT, forming T4 or T3, which remain covalently connected to the polypeptide backbone, while leaving a dehydroalanine at the donor site (73). Not every donor and acceptor tyrosine residue can be utilized to produce THs. A human TG molecule contains on average 2.28 molecules of T4 and 0.29 molecules of T3 (67).

#### TG storage and liberation

TG molecules – newly secreted into the lumen and thus remaining near the apical membrane where the I^-^ organification machinery is located – are the primary substrates of TPO. Freshly iodinated TG molecules, still soluble and adjacent to the apical surface, are also the first available for uptake by thyrocytes (67). If not taken up by thyrocytes, the iodinated TG molecules would proceed into the colloid where they are stored in a highly condensed, covalently cross-linked, multimerized globule (74–77). This unique storage mechanism makes TG highly enriched in the lumen, reaching a concentration as high as nearly 600 mg/ml in the human thyroid gland, and constituting >95% of the colloid protein content (67, 78–80). The turnover of this TG reservoir is very slow (81, 82). However, when the TG molecules in the colloid globule are needed to meet the TH demand, it is those on the outside of the globule, which are more recently iodinated, that are hydrolyzed quickly, rather than the ones packed inside, which are older and of greater iodothyronine content (83). Therefore, newer TG molecules are utilized first for TH production. This phenomenon is consistent with the “last-come-first-served” concept for the utilization of iodine – where the thyroid gland secretes recently formed organic iodine before it taps into the older ones (84).

Under circumstances such as iodine deprivation or TSH overstimulation, the colloid TG globule will be solubilized by proteinases and the solubilized TG will be taken up by thyrocytes (67). The proteinases expressed in the thyroid gland mainly include cathepsins B, D, H, K, L, and S (85). Cathepsins B, D, K and L are detectable in the follicle lumen or in association with the apical membrane (86–88). Cathepsins B and L in the lumen are involved in proteolytic solubilization of TG from covalently cross-linked TG globules (89). Trace amine-associated receptor 1 (TAAR1), a G-protein coupled receptor (GPCR), is located at the cilia of the apical plasma membrane of thyrocytes which extend into the follicle lumen. It can act as a sensor of the status of luminal TG. TAAR1 can co-localize with cathepsins B and L and regulate the proteolytic action of these cysteine cathepsins and internalization of TG (77).

#### TG uptake pathways

Soluble TG molecules of different maturity in iodination are taken up by thyrocytes through different mechanisms, including pinocytosis and endocytosis involving membrane receptors and related proteins (67, 90). Studies have shown that more recently synthesized TG molecules, which are low or yet to be iodinated, tend to bind to membrane receptors including megalin and ASGPR and are internalized along with receptor recycling, while highly iodinated and sialylated TG molecules preferentially undergo nonspecific fluid pinocytosis (68). TG molecules internalized *via* pinocytosis merge with lysosomes, in which T3 and T4 are proteolytically cleaved off of the TG backbone (67). TG molecules taken up *via* receptor-mediated mechanisms do not seem to be a significant source of T3 and T4 production. Megalin is a high-affinity TG receptor expressed on the apical surface of thyrocytes and it mediates the transcytosis of TG (67). It internalizes TG molecules with a low hormone content and the TG molecules so internalized are generally not available for degradation by the lysosomal pathway, but transported directly to the basolateral membrane and secreted into the blood circulation (91). ASGPR can also bind more recently synthesized, poorly iodinated and sialylated TG and route them away from the lysosomal pathway such that they serve as negative feedback signals to repress thyroid-specific TFs (TTFs) (68, 92). Flotillin, a lipid raft protein, also plays an important role in TG endocytosis. It can physically interact with endocytosed TG and mediate the negative feedback of TG on TTFs (90). ASGPR and *N*-acetylglucosamine receptor can recycle intracellular TG molecules of low hormone content back into the lumen (68, 93, 94).

### Intracellular TH liberation and release

Cathepsin K associated with the apical plasma membrane, particularly in active follicles, and luminal cathepsin L can act as endo- and exopeptidase to cleave soluble TG in the lumen, thereby liberating a limited amount of T4, which can be taken up by the thyrocytes (86, 88). However, the majority of T4 is produced intracellularly from pinocytosed TG. These TG molecules are degraded in lysosomes by cathepsins B, D, H, K, L, and S, where T3, T4, MIT, and DIT are exhaustively liberated (77, 95, 96). Proteolysis of TG releases 6 or 7 times more MIT and DIT than T3 and T4 (97). These MIT and DIT can be deiodinated by iodotyrosine dehalogenase (DEHAL), and the iodide released is recycled as a sustained source of intrathyroidal iodide for TH synthesis (98, 99). Intracellular T4 can be further deiodinated by DIO1 and DIO2 to produce T3 (100). T4 and T3 are then secreted into the bloodstream *via* TH transporters, including monocarboxylate transport MTC8 and MCT10, located on the basolateral plasma membrane (60). MCT8 favors T4 secretion over T3 secretion, as demonstrated by the higher-than-normal T3/T4 ratio in MCT8 KO mice and in patients with MCT8 mutation (101, 102). MCT10 has a similar effect on transporting T3 to MCT8 but is less active toward T4 than MCT8, as demonstrated in COS1 cells transfected with human MCT10 cDNA (103). The 2-3 amino acid difference in the substrate translocation channel of MCT8 and MCT10 appears to be responsible for the differential preference for T4 (104). While MCT10 does not appear to be expressed in the human thyroid gland, it is expressed in rodent thyrocytes, but still at a level much lower than MCT8 (105, 106). MCT10 seems to be dispensable as far as secreting TH is concerned since the thyroidal TH content is not elevated in MCT10 KO mice, whereas the thyroidal TH content accumulates to higher levels in MCT8 KO or MCT8/MCT10 double KO mice (101, 105). The lack of effect of MCT10 deficiency is not due to adaptive compensation by MCT8 because thyroidal MCT8 is not upregulated in MCT10 KO mice.

## TSH-stimulated signal transduction and transcriptional regulation in thyrocytes

### TSH activation of TSHR

**Figure 1:**
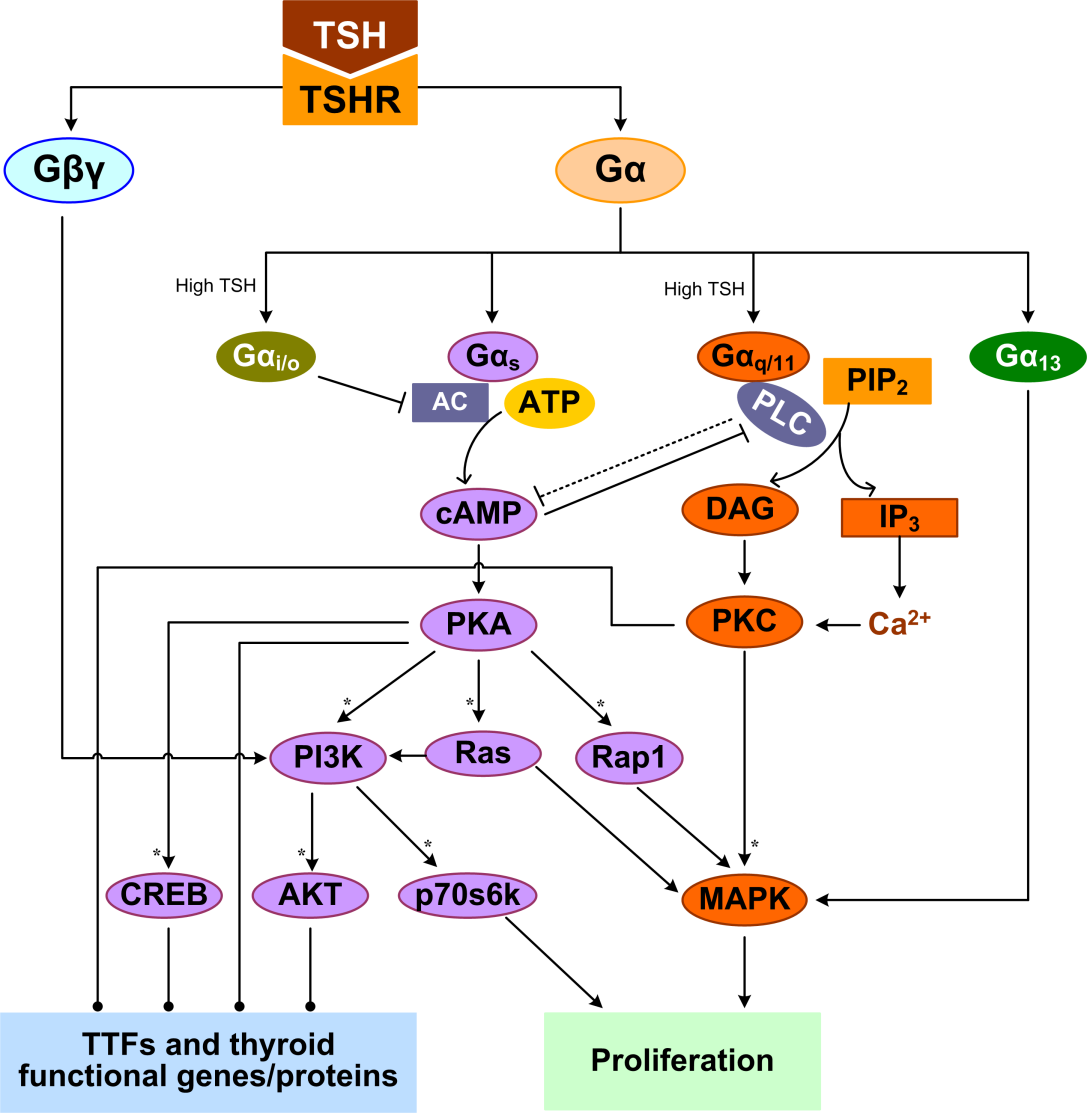
TSHR-mediated GPCR signal transduction pathways leading to TH synthesis and secretion and thyrocyte proliferation. Multiple feedforward motifs operate here. TSH activates TSHR leading to the dissociation of G proteins into Gα and Gβγ subunits. TSH can activate Gα_s_ to stimulate the production of cAMP, which then activates PKA. PKA can sequentially activate PI3K, p70s6k and AKT, as well as Rap1, Ras, and MAPK. High-concentration TSH can activate Gα_q/11_ and Gα_i/o_. Gα_q/11_ can activate PLC to convert PIP_2_ into DAG and IP_3_, which activate PKC and PKC activates MAPK. Both Gα_i/o_ and PLC inhibit the cAMP/PKA pathway, which can in turn inhibits PLC/PKC. Gα_13_ can activate the MAPK pathway, and Gβγ activates the PI3K/AKT pathway. MAPK and p70s6k can stimulate DNA synthesis and proliferation of thyrocytes. By regulating thyroid specific functional genes directly or through TTFs, PKA, AKT, PKC, and CREB regulate TH synthesis and secretion. Colors of molecule blocks are arbitrary. Solid lines: direct regulation, dotted lines: direct regulation not established, solid arrow heads: activation, blunted arrow heads: inhibition, dotted arrow heads: activation or inhibition, open arrow heads: mass flux, asterisk: phosphorylation. Unless otherwise specified, similar color scheme and denotations apply to all other figures.

TSH is the most important regulator of TH synthesis and secretion by the thyroid gland. The effects of TSH are mediated by the thyrotropin receptor (TSHR) which belongs to a family of GPCRs and is located at the basolateral plasma membrane of the thyrocytes (107). TSHR is a protomer composed of an A subunit which is a large extracellular domain, and a B subunit which comprises a 7-transmembrane-domain segment and an intracellular domain (108). Both subunits originate from a single peptide after cleavage but remain linked through disulfide bonds (109). TSH signaling may require the TSHR protomers to form a homodimer or higher-order oligomer (110, 111). Activation of TSHR leads to dissociation of G proteins into the Gα and Gβγ subunits which in turn trigger several canonical signal transduction cascades (112). TSHR can potentially couple to all four Gα protein families (Gα_s_, Gα_q/11_, Gα_i/o_, and Gα_12/13_) to activate several kinases that collectively regulate thyrocyte proliferation and TH synthesis and release (**Figure 1**) (113–115). The Gα_s_ and Gα_q/11_-induced signaling pathways are of the most importance in the thyrocytes.

### TSHR-activated signal transduction

TSH can differentially activate Gα_s_ and Gα_q/11_ in a concentration-dependent manner (116). The differential TSH sensitivity of the two pathways has been reported in both human thyrocytes and COS-7 and CHO cell lines transfected with human TSHR, suggesting it is an intrinsic property of the receptor itself (117–119). Low-concentration TSH binds to the high-affinity binding site on one of the protomers of the TSHR homodimer, and preferentially activates Gα_s_ to stimulate cyclic adenosine monophosphate (cAMP) accumulation, which then activates protein kinase A (PKA) (120). High-concentration TSH binds to both the high- and low-affinity protomers, activating both Gα_s_ and Gα_q/11_ to increase cAMP production and activate PKC, respectively (120). It was reported that it takes 10 times higher concentrations of TSH to activate Gα_q/11_ and the downstream phospholipase C (PLC) and inositol-1,4,5-trisphosphate (IP_3_) than to activate cAMP in human thyroid slices (117).

The TSH-stimulated thyroid response *via* Gα_s_ is believed to be a “long-term effect”, involving both TH biosynthesis and thyrocyte proliferation. A major target of PKA is the cAMP response element-binding protein (CREB) and its phosphorylation activates its transcriptional activity to induce a suite of downstream genes involved in TH synthesis, which are detailed in the sections below (121, 122). PKA signaling stimulates thyrocyte proliferation through several pathways. PKA can phosphorylate and activate small GTPase Rap1 to induce G1/S entry, as observed in PCCL3 cells (123). In FRTL-5 cells, TSH (cAMP) can amplify the activation of the PI3K and MAPK pathways and DNA synthesis elicited by IGF-I (124). In WRT cells, by activating cAMP and PKA, TSH can independently activate Ras and PI3K pathways and DNA synthesis (125–127). TSH, acting through cAMP, activates p70 ribosomal S6 kinase (p70s6k) that is required for TSH-stimulated DNA synthesis and proliferation in thyrocytes (128). Thus, the physiological function of cAMP signaling activated *via* Gα_s_ is to promote TH synthesis, thyrocyte growth and proliferation.

TSHR coupled to Gα_q/11_ can activate PLC to convert PIP_2_ into DAG and IP_3_. IP_3_ stimulates the release of Ca^2+^ from ER into the cytoplasm and activates, along with DAG, the PKC pathway. The effects of PKC on thyrocytes can be both stimulatory and inhibitory, depending on the signaling duration and cellular endpoints. PKC acts as a negative regulator of TH synthesis and secretion by inhibiting iodide organification (129). Preferential activation of the Gα_q/11_/PKC pathway by using a specific small molecule also inhibited TSH-stimulated proliferation of FRTL-5 cells (114). For the stimulatory response mediated by Gq/PKC, cathepsin B activity and TH-generating TG degradation were increased after 1-2 h of TSH treatment (130). Gq may be involved in the cleavage of T3 and T4 from iodinated TG by cathepsins through calcium signaling to liberate and release TH quickly in short term (77). PKC could induce DUOX2 phosphorylation and its H_2_O_2_-generating activity (59). Iodine organification and TH secretion in response to TSH are severely reduced in thyrocyte-specific Gα_q/11_-deficient mice which often develop hypothyroidism after birth (131). TSH or goitrogenic diet-stimulated proliferative thyroid response is also lacking in these mice. Besides, overexpression of atypical PKC-ζ in WRT cells induced TSH-independent DNA synthesis and cell proliferation through a p42/p44 MAPK-dependent pathway but had no effect on TG expression (132).

Like in many other types of cells (133–136), there exist cross-talks between the cAMP/PKA and PLC/PKC pathway in thyrocytes. Elevated cAMP levels can inhibit PLC activity (137), and PLC/PKC in turn suppresses the cAMP/PKA pathway, despite that the mechanism of suppression remains to be identified (114). At high TSH concentrations, TSHR can also activate Gα_i/o_, which inhibits adenylate cyclase activity to decrease cAMP production (138, 139). TSH can promote MAPK activation in human thyrocytes *via* a Gα_13_-dependent mechanism (113, 140). Besides, Gβγ released by TSHR activates the PI3K/AKT pathway (112). In summary, TSH stimulates multiple G protein-mediated signal transduction pathways, which cross-talk or converge at various levels of the signaling cascade. These cross-regulations can be either stimulatory or inhibitory, forming tiered, coherent or incoherent feedforward motifs (**Figure 1**).

### Feedforward regulation of TTFs

Through TSH-stimulated, GPCR-mediated signal transduction that culminates in PKA and PKC activation, a group of TTFs are activated. Four TTFs are expressed in differentiated thyrocytes: TTF1 (also known as NK2 homeobox 1, NKX2-1), TTF2 (also known as fork head box protein E1, FOXE1), paired box gene 8 (PAX8), and hematopoietically-expressed homeobox protein (HHEX) (141). These TTFs play critical roles in thyroid differentiation and also coordinately regulate the suite of functional genes involved in TH synthesis (142). Together with CREB, they form a complex transcriptional network, as detailed below (143).

**Figure 2:**
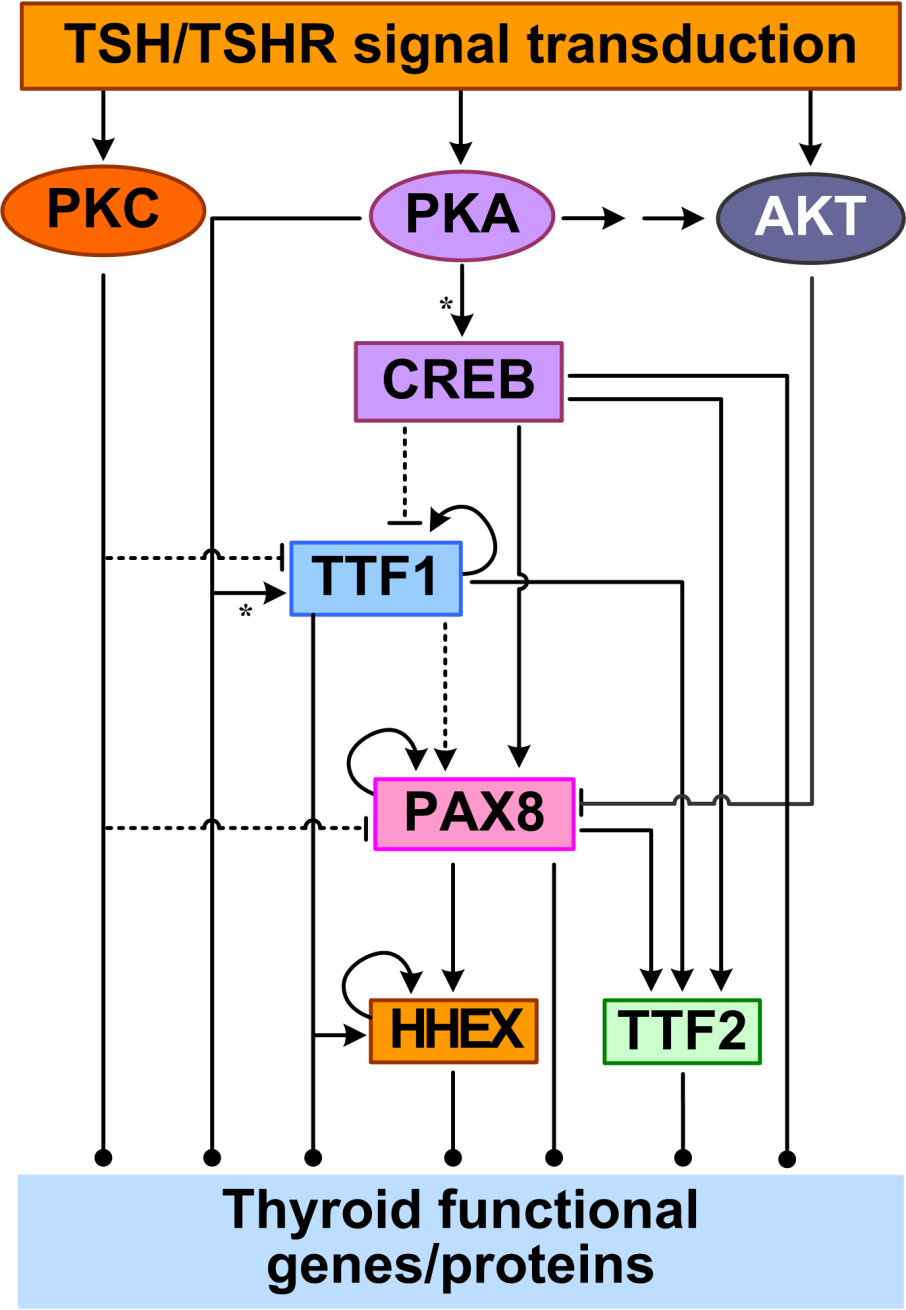
Feedforward and autoregulation of TTFs. CREB acts as a TF to repress TTF1 and induce PAX8 and TTF2 transcription. PKA can phosphorylate TTF1 to upregulate its transcriptional activity. PKC inhibits TTF1 and PAX8 expression, and AKT inhibits PAX8 by promoting its nuclear exclusion. TTF1 upregulates the expression of the other three TTFs. PAX8 also upregulates HHEX and TTF2. TTF1, PAX8, and HHEX also positively regulate their own expression. Asterisk: phosphorylation.

Feedforward appears to be the primary structure of the TTF regulatory network, in addition to autoregulatory loops (**Figure 2**). TTF1 are at the top of this core transcriptional network. With a TTF1-binding site identified *in silico* in the promoter of PAX8, TTF1 may regulate PAX8 gene expression (144). TTF1 and PAX8 appear to act together to co-regulate many downstream genes including HHEX and TTF2. The partnership between TTF1 and PAX8 may be through a couple of mechanisms. First, TTF1 and Pax8 are able to interact directly, as demonstrated in rat PCCl3 thyrocytes *in vitro*, thus they may form a functional protein complex driving synergistic transcriptional activation of target genes such as *TG* (145). Second, the DNA binding sites of TTF1 and PAX8 often colocalize in the promoters of target genes (143). Functional binding sites for both TTF1 and PAX8 have been found in the *HHEX* promoter, and HHEX expression can be induced (146, 147). The HHEX promoter activity could be increased by 3~4 fold by TTF1 in rat FRTL-5 thyroid cells (146) and by PAX8 in transfected Hela cells (147). In human thyroid tissues, the mRNAs of TTF1 and HHEX were found to be positively correlated (146) and so were the mRNAs of PAX8 and HHEX (147). Another common inducible target of TTF1 and PAX8 is TTF2 (148, 149). PAX8 can bind to one site in the 5′-flanking regions of the *TTF2* gene and activate its transcription in FRTL-5 cells (150). In a microarray study using PCCl3 cells, TTF2 was downregulated significantly in the presence of a TTF1 inhibitor (151). Taken together, starting from TTF1, mediated through PAX8, and ending on HHEX and TTF2, at least two coherent feedforward motifs are formed among the four TTFs (**Figure 2**). Lastly, positive autoregulatory loops also exist for TTF1 (152), PAX8 (150) and HHEX (146).

The core network of the four TTFs receives input signals from the basolateral plasma membrane through multiple signal transduction pathways initiated by TSH. In the cAMP/PKA/CREB pathway, CREB acts as a TF to directly regulate the gene expression of PAX8, TTF2, and TTF1. TSH can stimulate PAX8 mRNA and protein expression, likely *via* the CREB binding sites in the PAX8 gene promoter, as demonstrated in PCCl3 and FRTL-5 cells (144, 153). Although no CREs have been determined in the TTF2 gene promoter, in FRTL-5 cells it was found that TSH-stimulated upregulation of TTF2 mRNA levels appears to be mediated by the cAMP/PKA/CREB pathway (154). In contrast to the CREB-mediated upregulation of PAX8 and TTF2 by TSH, TSH downregulates TTF1 mRNA and protein by inhibiting the promoter activity and transcription of TTF1 in FRTL-5 cells (155, 156). The inhibition appears to require the presence of insulin signaling and can be reproduced by forskolin, strongly suggesting the involvement of cAMP/PKA/CREB pathway (155). However, there is no CREB/CREM consensus sequence identified within 2.5 kb of the 5′-upstream regions of TTF1 gene (155). With CREB added to the top of the TTF network, more feedforward motifs are formed, including incoherent ones through the inhibition of TTF1 by CREB (**Figure 2**).

Other signal transduction pathways activated by TSH also regulate the TTF network. Huang et al. reported that activating the PLC/PKC pathway by using phorbol esters inhibited TTF1 and PAX8 expression in human Nthy-ori-3-1 thyrocytes (157). The Gβγ-activated PI3K/AKT signaling pathway can cause PAX8 exclusion from the nucleus and thus inhibit PAX8-mediated NIS transcription in PCCl3 cells (112).

### Feedforward and feedback regulation of thyroid functional genes

**Figure 3:**
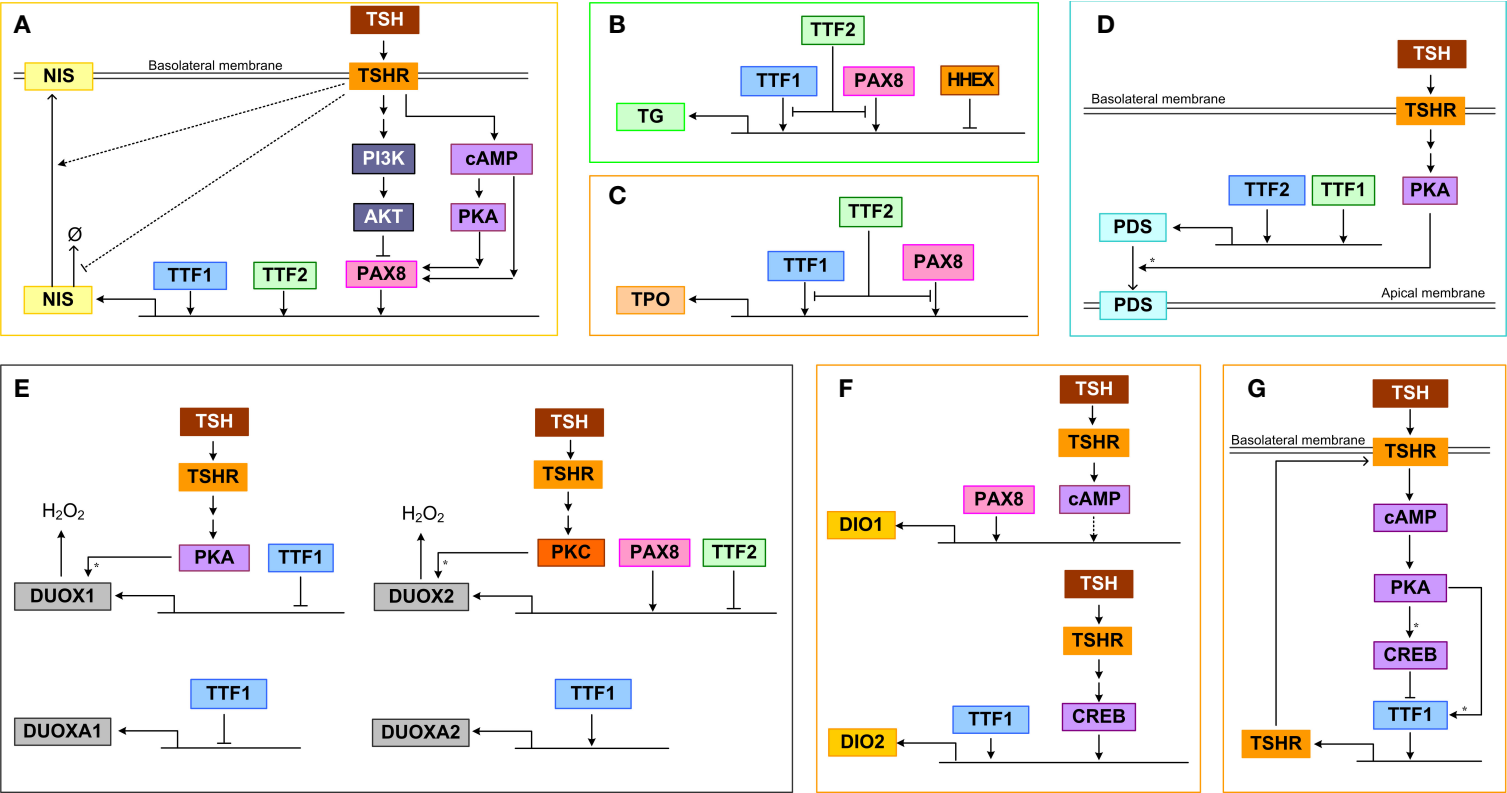
Transcriptional and posttranscriptional regulation of thyroid functional genes. **(A)** NIS, **(B)** TG, **(C)** TPO, **(D)** PDS, **(E)** DUOX(A)1/2, **(F)** DIO1/2, **(G)** TSHR. Ø denotes degradation. For molecular details see the main text. Asterisk: phosphorylation.

Binding of TSH to TSHR activates signal transduction pathways that regulate TTF activities, which in turn control the gene expression of many thyroid-specific functional proteins, including NIS, pendrin, TG, TPO, DUOX(A)1/2, DIO1 and DIO2, that participate in the synthesis and secretion of THs (158, 159). The TTFs, mainly TTF1, TTF2 and PAX8, as well as the TFs terminally activated by the signal transduction pathways, such as CREB, converge on the cis regulatory elements of the above target genes and regulate their expression in a feedforward fashion (**Figure 3**).

#### NIS

TTF1, TTF2, and PAX8 can bind to the upstream enhancer and promoter regions of *NIS* and stimulate its transcriptional activity (**Figure 3A**). TTF1 was demonstrated to bind to a DNA sequence about 200 bp upstream of the transcription start site of *NIS* in rats and induce promoter activity (160). In a genome-wide screening study in *TTF2* knockdown cells, it was discovered that TTF2 can bind to the upstream enhancer region close to an NF1/CTF binding site and these two TFs cooperate to upregulate NIS-promoter activity (161). PAX8 can bind to two enhancers that are upstream NIS promoter to induce NIS transcription, and mediate the Gα_s_/cAMP-stimulated NIS induction in both a PKA-dependent and independent manner (162). In primary human thyrocytes, knockdown of *TTF1*, *TTF2*, *PAX8* but not *HHEX* genes markedly inhibited TSH-stimulated *NIS* mRNA expression (163). For the PKA-dependent response, a CRE-like binding site seems to be required. Conversely, the TSH-stimulated Gβγ/PI3K/AKT pathway can somehow induce nuclear exclusion of PAX8 and repress PAX8 binding to the NIS enhancers, thus inhibiting NIS transcription (112). Through activating the PI3K/AKT pathway, IGF-1 exerts a synergistic effect on TSH-stimulated NIS expression, which is mediated by ERK1/2 phosphorylation (164). In addition to transcriptional regulation, NIS also appears to be regulated by TSH posttranslationally. It has been demonstrated that TSH can increase the half-life of NIS protein and plasma membrane translocation, which may be mediated by TSH-stimulated NIS phosphorylation (165).

#### TG

The transcriptional activity of the TG promoter can be activated by both PAX8 and TTF1 (**Figure 3B**). This was demonstrated by reporter assays in COS-7 and HeLa cells which were co-transfected with a vector containing a 202 bp fragment from the human TG 5’-flanking region, including the promoter sequence and the transcriptional start site, and vectors containing the cDNAs encoding human TTF1 and PAX8 (166). PAX8 and TTF1 are able to interact directly *in vitro* and form a functional complex *in vivo* responsible for the synergistic transcriptional activation of the TG promoter (145). Overexpression of PAX8 in FRTL-5 cells increased TG promoter activity in a reporter assay (153). However, in the same study TTF1 was found to compete with PAX8 for binding to the promoter and plays a minor inhibitory role. TTF2 could interfere with the binding of TTF1 to the TG promoter, inhibiting its transcriptional activation (167). It was suggested that the promoter-specific inhibitory activity of TTF2 is mediated by a C-terminal domain that interacts with a co-factor involved in TTF1 and PAX8-mediated target gene transcription (168). In primary human thyrocytes, knockdown of *TTF1*, *TTF2*, *PAX8* but not *HHEX* genes markedly inhibited TSH-stimulated TG mRNA expression (163). HHEX can bind to the TG promoter and function as a repressor to abolish the activating effects of both TTF1 and PAX8 in FRTL-5 cells (159). The HHEX mRNA and protein expression can be downregulated by TSH under certain conditions and this disinhibition may permit TG transcription by TTF1 and PAX8 (159).

#### TPO

TTF1 and PAX8 can activate the transcription of TPO by binding to specific DNA binding sites that overlap each other in the promoter region (**Figure 3C**) (169). Under TSH and cAMP activation, TTF2 can directly bind to the TPO promoter in rat FRTL-5 thyroid cells (170), which may interfere with the binding of TTF1 and PAX8 to the promoter, inhibiting the transcriptional activation of TPO (167). In primary human thyrocytes, knockdown of *TTF1*, *TTF2*, *PAX8* but not *HHEX* genes markedly inhibited TSH-stimulated TPO mRNA expression (163).

#### PDS

TTF1 is the main transcriptional activator of the PDS promoter, controlling the expression of PDS in thyroid cells (**Figure 3D**) (171). Between nucleotides (nt) -1946 and -1938 and between nt -1942 and -1933 on the PDS (*SLC26A4*) gene promoter exist putative binding sites of TTF1 and TTF2 respectively which are essential for the activity of the PDS promoter, as demonstrated by mutation analysis in human thyroid follicular carcinoma (LA2) cells (172). TSH-stimulated PKA can phosphorylate PDS to promote its membrane translocation, leading to increased iodide efflux into the follicular lumen (173, 174).

#### DUOX(A)1/2

PAX8 can bind to the 5′-flanking region of the *DUOX2* gene to activate its transcription (**Figure 3E**) (150). TTF2 can also bind to the promoter of *DUOX2* but repress its transcription (161). PKC can phosphorylate DUOX2 and induce its H_2_O_2_-generating activity, while PKA-mediated phosphorylation of serine 955 of DUOX1 can stimulate its activity (59). When TTF1 was inactivated, the expression of DUOXA2 was downregulated, while the expression of DUOX2 remained unchanged, and both DUOX1 and DUOXA1 were upregulated. However, in transient co-transfection experiments, the cloned proximal promoter sequences from the DUOX genes were unresponsive to TTF1 (175).

#### DIO1/2

The promoter of the *DIO2* gene contains both CREB-binding site and TTF1 binding site, and TSH and TTF1 were shown to upregulate DIO2 activity (**Figure 3F**) (176, 177). In primary human thyrocytes, knockdown of *TTF1*, *TTF2*, *PAX8* but not *HHEX* genes markedly inhibited TSH-stimulated DIO2 mRNA expression (163). Abolishing PAX8 downregulated DIO1 expression in thyroid cells, suggesting that PAX8 activates DIO1 transcriptionally, which is likely mediated by the selenocystein insertion sequence in the 3’ untranslated region of the *DIO1* gene (178). In addition, DIO1 is upregulated by TSH *via* cAMP, however weather CREB is directly involved is unclear (179). The upregulation of DIO1 and DIO2 by TSH is important in establishing the intrathyroidal TSH-T3 shunt pathway (180) to preferentially stimulate T3 production (181). TSHR-mediated upregulation of DIO2 contributes significantly to the relative increase in intrathyroidal T4-to-T3 conversion and thus T3 production and higher circulating T3/T4 ratio in patients with Graves’ disease and toxic adenomas (182, 183). Plasma free T3 concentration has a clear circadian pattern with a periodicity that lags behind TSH (184, 185), and this periodic rhythm of T3 is partly attributed to the thyroidal T4-to-T3 conversion driven by TSH which itself is under circadian control (180).

#### TSHR

TSHR is under autoregulatory feedforward and feedback control by TSH (**Figure 3G**). In the “minimal” promoter region of the TSHR gene, there exists a TTF1 binding site, which can mediate its transcriptional activity (186, 187). In FRTL-5 thyroid cells, TSH treatment first upregulated TSHR mRNA moderately in the initial 2 hours and then downregulated it by 75%, and the mRNA recovered in 6 days after TSH withdrawal (188, 189). Treatment with cAMP analogs or activators produce a similar effect, and it seems that low cAMP tends to increase but high cAMP decreases TSHR expression. The TTF1 binding to TSHR promoter shows a similar biphasic trend (190). The initial increase is likely caused by phosphorylation of TTF1 by PKA, which promotes its binding to TSHR promoter and enhances the transcriptional activity (190). However, the subsequent transcriptional repression of TTF1 by CREB is responsible for the downregulation of TSHR. The TTF1 activity on the *TSHR* gene appears to require the presence of a CRE-like sequence near the TTF1 binding site in the minimal TSHR promoter (186) and the negative effects also require the presence of insulin (156). In primary human thyrocytes, knockdown of *TTF1* and *TTF2* but not *HHEX* and *PAX8* genes markedly inhibited TSH-stimulated TSHR mRNA expression (163). While likely important for TSHR homeostasis and receptor desensitization, the positive and negative regulations of TSHR by TSH and cAMP form an incoherent feedforward control. This structure explains the transient upregulation of TSHR and may also function to speed up the thyroid response or optimize the response to pulsatile TSH. Changes in TSHR expression in turn affect activation of PKA by TSH forming a positive or negative feedback loop, depending on the direction in which TSHR is regulated.

#### ANO1

The transcription and accumulation of ANO1 at the apical membrane of thyroid follicles were stimulated by TSH in rats (54). The mechanisms underlying ANO1 activation and modulation are just beginning to emerge. It has been shown that calmodulin, protons, cell volume and thermal stimuli can regulate the channel activation (191, 192), and PIP_2_ regulates the activation and desensitization of ANO1 (193, 194)

#### SLC26A7

The TSH/cAMP signaling cascade suppresses the mRNA and protein expression of SLC26A7 in FRTL-5 thyrocytes, and this suppressive effect may be mediated, at least in part, by downregulation of TTF1 expression under TSH. In addition, TSH induces the translocation of the SLC26A7 protein to the plasma membrane, which may be attributed to the phosphorylation of the STAS domain of SLC26A7 by the TSH signaling cascade (195).

### Regulation between MCT8/10 and TSHR

The release of THs from TG into the blood circulation can be rapidly regulated, involving direct interaction between MCT8 and TSHR (196). The two can form heterodimeric complex which directly modulates the selectivity of TSHR-mediated signal transduction pathways. MCT8 profoundly reduces the capacity of TSH to induce Gα_q/11_-mediated signaling at TSHR, without affecting Gα_s_-mediated cAMP accumulation (196). Since Gα_q/11_ is involved through calcium signaling in the cleavage of T3 and T4 from TG by cathepsins and their release as an acute effect (77, 130), MCT8, by heterodimerizing with TSHR and interfering with Gα_q/11_ signaling, may inhibit T3/T4 release, while the long-term stimulatory effect of TSH mediated by Gα_s_ is spared. Besides, MCT10 plays an important role in maintaining TSHR at the basolateral plasma membrane of the thyrocytes. In murine models lacking MCT10, the localization of TSHR is restricted to vesicles (197).

### Biphasic response to TSH

The specificity of Gα protein activation appears to depend on the level of TSH, which can biphasically regulate thyrocyte functions (198). In primary human thyrocytes, TSH induced bell-shaped dose-response of HHEX, TG, NIS, TPO, DIO2, and TSHR mRNA expression and secreted TG protein (163, 198). The TSH concentration associated with the peak response is about 10 mU/mL. NIS protein also exhibits a bell-shaped response to TSH (164). The exact mechanism on how these nonmonotonic responses to TSH arise is not entirely clear. It may involve differential regulation of TTFs by TSH. In 2D cultured primary human thyrocytes, TSH concentration-dependently inhibited TTF1, TTF2, and PAX8 mRNA expression modestly (163). In another study also using primary human thyrocytes, TSH at 1 or 5 mU/mL stimulated TTF1, TTF2, and PAX8 mRNA expression while in 3D culture these three genes did not respond to TSH stimulation (199). As described above, the TSH-stimulated signal transduction pathways can form incoherent feedforward motifs to differentially regulate TTFs and thyroid functional genes, and incoherent feedforward is well known for generating nonmonotonic dose responses (200). The biphasic responses may originate from the biphasic cAMP activation by TSH as demonstrated *ex vivo* in human thyroid tissues (201). In primary human thyrocytes and HEK 293 cells expressing human TSHR, it has been recently demonstrated that while the stimulatory phase of the bell-shaped cAMP response depends on Gα_s_, the inhibitory phase requires sufficient TSHR on the cell surface to support homodimer formation and is mediated *via* Gα_i/o_ (138, 139, 202). Therefore, the incoherent feedforward motif formed by Gα_s_ and Gα_i/o_ (**Figure 1**) is responsible for the biphasic cAMP response and, likely, similar downstream gene expression responses.

The functional significance of this biphasic response of the thyroid to TSH is not clear. Biphasic response has been proposed as part of a mechanism for robust tissue size control against mutations in endocrine systems (203), For example, in the feedback regulation of blood glucose homeostasis, the biphasic proliferative response of pancreatic β cells to glucose is proposed to prevent the invasion by mutant β cells that have markedly gained sensitivity to glucose. By a similar token, the biphasic response to TSH may play an important role in the homeostatic control of the thyroid gland size against gain-of-function mutations.

### Summary

In summary, starting at TSHR, the TSH signal is transmitted through a complex cascade of signal transduction, transcriptional and posttranslational regulations that diverge and converge at different nodes and layers of the cascade. The divergence and convergence of these regulations primarily form interconnected, coherent and/or incoherent feedforward controls. Feedforward controls are well known for their capability to differentiate transient vs. persistent signals, generate sign-sensitive response delay or acceleration, and produce nonmonotonic dose-response (200). As described above, the TSHR-initiated feedforward signaling cascade has manifested some of these features, including transient transcriptional induction of TSHR and the biphasic response to TSH. However, the complexity does not stop here. As revealed below, a number of negative feedback regulatory pathways also operate on top of the feedforward backbone, providing important functions to thyroid homeostasis.

## The high-iodine effects

While dietary iodine deficiency can result in goiter and hypothyroidism due to the essential role of iodine for TH synthesis, interestingly, excess iodine intake can also lead to inhibition of thyroid functions. Several mechanisms appear to be at play, affecting thyroidal iodide organification, uptake, and recycling, as well as TH liberation, release, and deiodination.

### The Wolff-Chaikoff phenomenon

In the 1940s, Wolff and Chaikoff reported that when large amounts of iodide were administrated intraperitoneally in rats, after an initial increase in the intrathyroidal iodide store, the organification of iodine and thus TH synthesis were inhibited in a couple of hours (204, 205). The inhibitory effect on iodine organic binding, as estimated by the thyroidal I^131^ accumulation, was also reported in euthyroid and thyrotoxic men, hours after administration of high iodine (206). This phenomenon was named afterward as the Wolff-Chaikoff effect. The inhibition is transient, lasting approximately 26-50 h in rats, followed by adaption and escape of the thyroid from the inhibition despite ongoing iodine excess – the organification of intrathyroidal iodide resumes and TH synthesis returns to near normal levels (207, 208).

### The Plummer effect

High iodine also inhibits TH secretion. In humans, serum T4 and T3 quickly decreased after high iodine intake and the decrease could persist for days or even weeks as long as the serum iodide remained high (209–211). The lowered serum THs and secondary increase in TSH can be reversed upon iodine excess removal. The decrease in serum TH levels is attributed to inhibition of TH secretion not synthesis because antithyroid drugs did not produce a similar effect as high iodine intake in comparable period, and the inhibition of iodide organification (and thus TH synthesis) resulting from the acute Wolff-Chaikoff effect is expected to be transient (210). The inhibition of TH secretion by high iodine was named the Plummer effect after the endocrinologist Dr. Henry Plummer, who discovered that high-dose iodine can be used to treat Graves’ disease and reduce postoperative death from thyroid surgery (212, 213). The Plummer effect is more prominent in hyperthyroid patients but also occurs, to a less degree, in euthyroid individuals (214). The Plummer effect seems to be more variable in animal studies. In Wistar rats, high NaI administered daily induced decrease in serum T4 and to a lesser extent in T3 after one day, and by day 6 T4 was normalized with T3 still somewhat lower than normal (102). In another study using Wistar rats, no changes in THs and TSH were observed (215). In Sprague Dawley (SD) rats exposed to NaI in drinking water, T3 and T4 decreased on day 1 but fully recovered in 6 days (216). Therefore, the Plummer effect in rodents may not be as persistent as in humans.

It is worth noting that the literature often confuses the Plummer effect as part of the general Wolff-Chaikoff phenomenon. But as described above, the latter involves the inhibition of iodide organification, whereas the former involves the inhibition of TH secretion (217, 218). Correspondingly, there are mechanistic differences between the two, as further detailed below.

### Feedforward and feedback regulations mediating the high-iodine effects

**Figure 4:**
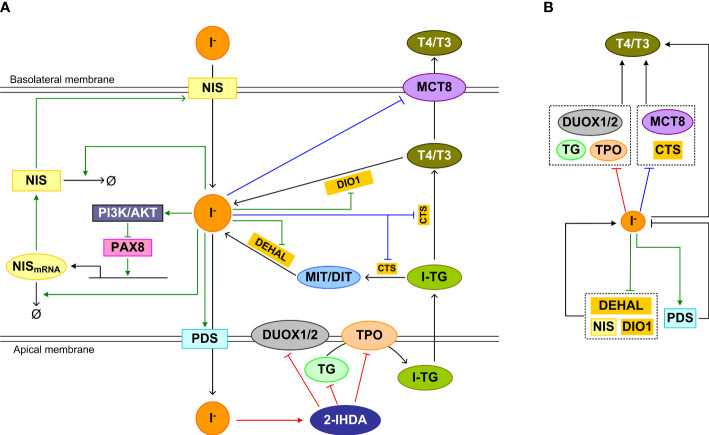
Feedforward and feedback regulations underpinning the high-iodine effects. **(A)** Inhibition mechanism of Wolff-Chaikoff effct: The intermediate product iodolipid, 2-IHDA, formed in the follicular lumen by high iodide, inhibits DUOX1/2, TPO, and TG expression and/or activity acutely, reducing iodide organification and TH synthesis. Inhibition mechanism of Plummer effect: Iodine excess also inhibits iodinated TG (I-TG) proteolysis and MCT8 expression, which reduces TH liberation and release. Wolff-Chaikoff escape mechanism: chronically, iodine excess inhibits NIS expression, promotes NIS mRNA and protein degradation, upregulates and stabilizes PDS, and decreases DEHAL and DIO1 mRNA levels and activities to normalize iodide level in thyrocytes and eventually TH synthesis and release. **(B)** Concise view of **(A)** illustrating incoherent feedforward regulation of T4/T3 associated with the Wolff-Chaikoff and Plummer inhibitions, and negative feedback regulation of intracellular iodide associated with Wolff-Chaikoff escape. Red arrows: pathways of Wolff-Chaikoff inhibition; Blue arrows: pathways of Plummer inhibition; green arrows: pathways of Wolff-Chaikoff escape.

It is believed that the inhibitory effect of high iodine is an effective homeostatic means by which the thyroid gland prevents the large quantities of iodide that enters thyrocytes from causing excessive TH synthesis and secretion. The inhibition involves blockade of multiple molecular events in the sequential processes of TH synthesis and secretion, including iodide organification, proteolysis of iodinated TG to liberate T4 and T3, and release of T4 and T3 into the blood stream (**Figure 4A**).

#### Wolff-Chaikoff inhibition mechanism

The Wolff–Chaikoff effect requires a high intracellular concentration (≥ 1 mM) of iodide (219). When the intrathyroidal iodide fell well below 1 mM, organic iodination resumes in TSH-stimulated thyroid glands (220). Since iodide is required for TH synthesis and high iodide inhibits TH synthesis through the mechanisms detailed below, it follows that iodide regulates TH synthesis in an incoherent feedforward manner (**Figure 4B**, top half involving red line).

Iodolipid, such as 2-iodohexadecanal (2-IHDA), is believed to play a major role in mediating the Wolff-Chaikoff inhibitory effect. High iodide inhibits H_2_O_2_ production in cultured thyroid cells (221), which is mediated by 2-IHDA (222, 223). The inhibitory action may involve a direct effect of 2-IHDA on the membrane NOX activity (224). More recently, it was demonstrated that the decrease in H_2_O_2_ production by high concentrations of 2-IHDA is likely mediated by altered DUOX1/2 expression (225). 2-IHDA strongly inhibits other TSH-stimulated genes including TPO and TG in a concentration-dependent manner, which could be attributed to dysregulation of PAX8, TTF1, and TTF2 expression and promoter binding (225). Thyroidal TPO mRNA expression was observed to decrease after 1 day of acute *i.p.* administration of 2 mg KI in rats (216). Repeated oral-gavage administration of 1 mg/kg KI every 4 days for 8 days in rats also caused a decrease in TPO mRNA expression (215). The inhibition of expression of these thyroid specific genes could also be mediated by highly iodinated TG as detailed in the section of TG feedback below.

#### Plummer inhibition mechanism

Excess iodine lowers serum thyroxine quickly, suggesting that it blocks the secretion of preformed hormones stored in the follicular lumen (219). This is believed to result from reduction of the proteolytic cleavage of iodinated TG to liberate T4 and T3 and inhibition of TH secretion from the thyrocytes (226), which form another incoherent feedforward motif (**Figure 4B**, top half involving blue line). Reduced proteolysis of TG was found in Grave’s patients who received Lugol’s solution and were normalized to euthyroid state, compared with those who were not normalized by the Lugol’s solution treatment (227). Varying susceptibilities of iodinated TG to proteolysis have been reported in animal studies in response to high iodine. It was demonstrated in SD rats that the proteolysis of iodine-rich TG preferentially formed during high iodine diet was reduced (228). Also in SD rats, high iodine inhibited thyroidal cathepsin D activity (229). In another study, no effect on the proteolysis of TG by lysosomes was observed in rats, but guinea-pigs treated with excess iodide exhibited increased TG proteolysis for 3 days after which the effect was reversed to the other direction (230). No inhibition of TG proteolysis was observed *in vitro* with Wistar rat thyroids, but increased proteolysis was observed *in vivo* (231). Therefore, there exists species difference in the effect of high iodine on TG proteolysis and further studies are needed.

Lastly, iodide overload decreased MCT8 mRNA and protein expression in rats, thus reducing T4 and T3 secretion into the blood stream (102, 215). TPO activity appears to be required for the inhibition of MCT8 by high iodide load (102). In the study by de Souza et al, the downregulation of MCT8 was transient, which recovered by day 6. This transient nature of MCT8 downregulation may be responsible for the eventual normalization of serum TH levels in rats despite continuous exposure to high iodine (216).

#### Wolff-Chaikoff escape mechanism

The thyroid gland is an effective collector of iodine, containing about 10-15 mg of iodine in humans (232). It can handle different iodine loads efficiently to maintain intrathyroidal iodide homeostasis. The escape phenomenon seems to be a result of adaptation in response to high intracellular iodide load, involving negative feedback regulation of multiple processes that participate in the intracellular iodide turnover (**Figure 4B**, lower half). The main known mechanism mediating the escape is the downregulation of NIS and thus the decrease of iodide uptake. The iodide uptake capacity of thyrocytes is inversely associated with its serum concentration. The reduced iodide uptake through downregulation of NIS by high iodine is exploited to prevent radioactive iodide uptake by the thyroid gland in the event of nuclear accidents or wars (233). NIS is downregulated by high iodide through several mechanisms. First of all, high iodide inhibits the transcription of NIS (216, 234, 235). After exposure to excess iodide, the NIS mRNA level in rat thyroid cells decreased at 6 and 24 h, and the NIS protein level also decreased at 24 h (236, 237). In Wistar rats treated with KI for 8 days, NIS mRNA is downregulated continuously after day 1 (215). In SD rats exposed to high NaI, NIS protein was downregulated for 6 days (216). The repression is mediated by the activation of the PI3K/AKT pathway, which induces nuclear PAX8 exclusion and reduces the binding of PAX8 to the NIS upstream enhancer (237, 238). Second, high iodide resulted in shortening of the NIS mRNA poly (A) tail and caused the transcript to become more susceptible to degradation (239). The destabilization contributed to a shorter half-life of NIS mRNA and thus lower abundance (240). Last, high plasma iodide concentrations led to shortening of the half-life of NIS protein from 4 day to 24 h (216). The iodine organification activity of TPO appears to be essential for the blockade of iodide uptake elicited by iodine overload. When TPO was inhibited by methimazole, the inhibition of iodine uptake induced by high iodine was also abolished, suggesting that organified iodine is involved in the NIS-mediated escape from high iodine-induced thyroid inhibition (241, 242).

Upregulation of PDS may be involved in the escape process as well. As early as 30 min after acute iodide treatment, the PDS mRNA level was transcriptionally upregulated, which persisted for 48 h in rats (243). PDS mRNA upregulation was also observed in rats between 4-10 days with repeated daily oral gavage of KI (215). Iodide excess also reduced the degradation of PDS protein leading to an increase of the protein half-life (244). After 24 h of iodide treatment, the insertion of PDS into the plasma membrane was increased. The increased PDS density in the plasma membrane is expected to move more iodide out of the thyrocytes to the lumen, reducing its intracellular concentration.

The downregulation of DEHAL and DIO1 may also contribute to the escape from the Wolff–Chaikoff effect. Both acute and chronic iodide administration significantly decreased DEHAL and/or DIO1 mRNA levels and activities in the rat thyroid (245, 246). The downregulation would result in reduced recycling of iodine from MIT, DIT and THs, which may help to alleviate the situation of high intracellular iodide.

Taken together, the three different mechanisms, including the Wolff-Chaikoff inhibition effect, the Plummer effect, and the adaptive escape, work together to fend off the potentially harmful consequence of excess iodine load and ensure thyroid homeostasis.

## TG-mediated feedback regulation

### TG-mediated feedback regulation of thyroid genes and physiological significance

**Figure 5:**
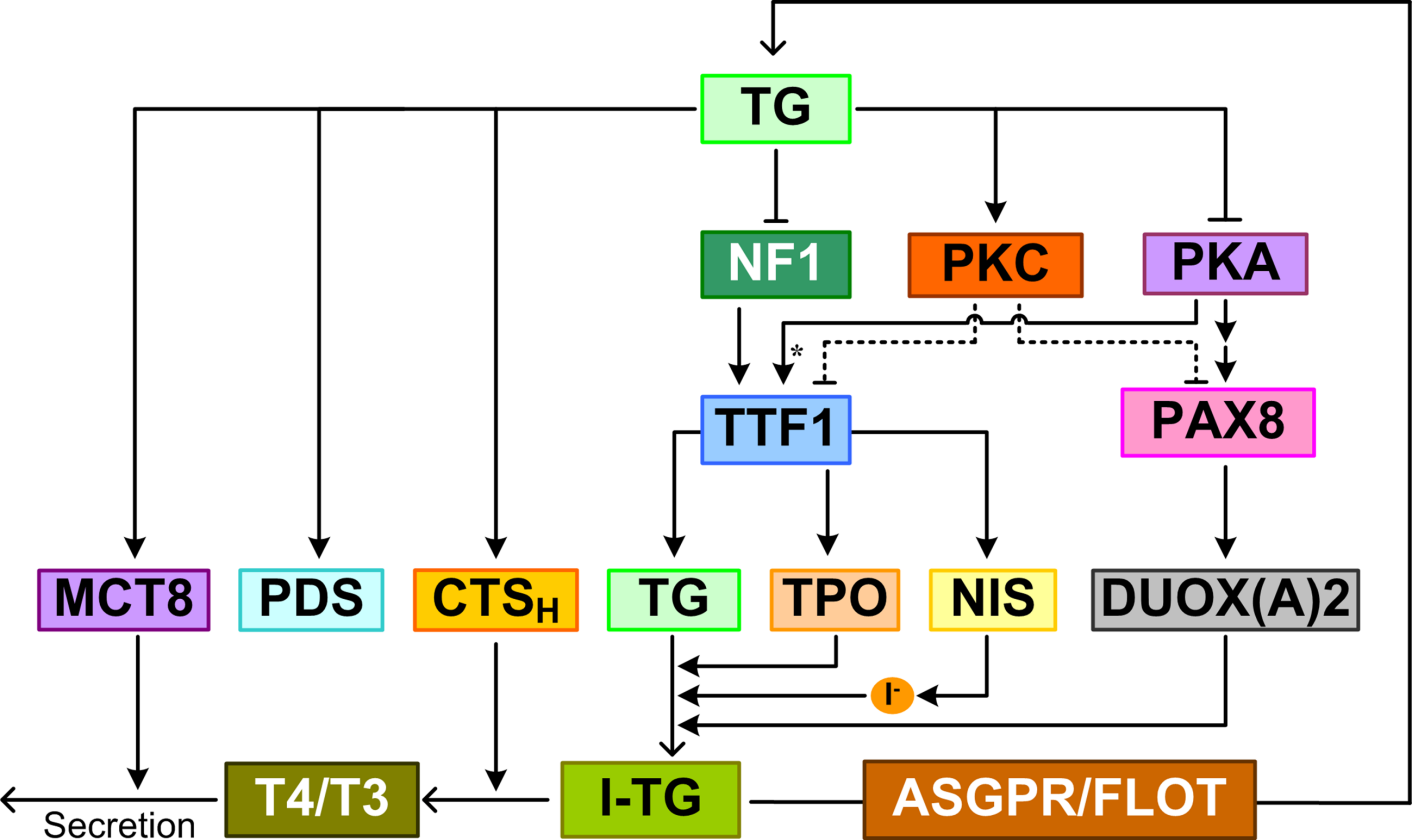
TG-mediated feedback. TG regulates TH synthesis and release by suppressing the expression of NIS, TG, TPO, DUOX(A)2 and upregulating the expression of PDS, MCT8, and CTS_H_ (cathepsin H). I-TG translocates from the follicular lumen into thyrocytes *via* ASGPR and FLOT. TG inhibits NF1 expression and activity to repress TTF1 gene expression. TG also represses TTF1 and PAX8 expression by augmenting PKC and suppressing PKA activities. Asterisk: phosphorylation.

TG is an intrinsic feedback suppressor on the effects of TSH in thyroid follicles (**Figure 5**). TG accumulated in the follicular lumen regulates TH synthesis by suppressing thyroid-specific gene expression in a concentration-dependent manner (247). Experimental evidence obtained in rat thyroid FRTL-5 cells showed that TG at physiological follicular concentrations suppressed the expression of key genes involved in TH biosynthesis, including TG itself, NIS, TPO, DUOX2, DUOXA2, TTF1, TTF2, PAX8, and TSHR (142, 247–250). A similar negative feedback regulation by follicular TG was also observed in primary cultures of normal human thyrocytes (251). The TG-mediated gene suppression eventually results in inhibition of TSH-stimulated iodide uptake and H_2_O_2_ generation (249, 250, 252).

Besides downregulating gene expression, TG can also upregulate the expression of some genes, including PDS (253). PDS expression was dramatically increased in cells exposed to low-level TG (249, 254). In FRTL-5 cells cultured with TSH and subsequently treated with 1 mg/ml TG, PDS mRNA was induced in 3 h and peaked at 24 h (249); when TG was removed from the culture medium at 24 h, PDS mRNA disappeared 6 h later. In FRTL-5 cells, bovine TG induced upregulation of cathepsin H mRNA, protein, and enzymatic activity (255). In addition, TG endocytosis promoted cathepsin H translocation into lysosomes where the proteinase degrades iodinated TG to release T4 and T3. A similar upregulation of MCT8 mRNA and protein was also observed in bovine TG-treated FRTL-5 cells (256). Unlike DUOX2 and its maturation factor DUOXA2, which were repressed by TG feedback, DUOX1 and DUOXA1 expression was largely unaffected by TG (257).

The functional purpose of the TG-mediated feedback is not entirely clear. With the exception of upregulation of PDS, it appears to inhibit processes leading to accumulation of iodinated TG, and promote processes of TG lysosomal degradation and TH release (255). Therefore, the TG-mediated feedback may function to maintain the homeostasis of iodinated TG, rather than THs.

### Effectors of TG-mediated feedback

The feedback effect of TG appears to depend on its degree of iodination. The expression of NIS, TG, and TPO mRNAs was found to decrease significantly in follicles reconstituted from primary pig thyrocytes and treated with a high dose of iodine for 3 days compared with the control (258). The inhibitory effect of high iodine can be blocked by MMI, an inhibitor of TPO, suggesting that iodinated TG mediates the inhibition of gene expression. Although a direct, TG-independent Wolff-Chaikoff effect cannot be ruled out here, further experiments showed that compared with lowly iodinated TG, highly iodinated TG extracted from the reconstituted follicles inhibited the expression of the above genes in primary pig thyrocytes monolayer culture (258). Highly iodinated TG suppressed TTF1 and PAX8 expression at both mRNA and protein levels, which is likely mediated by augmented PKC and suppressed PKA activities (157).

TG control of thyroid function is an “apical” phenomenon, i.e., it requires receptors in direct contact with high-concentration TG in the follicular lumen. Receptors that were proven to bind TG and transduce the effect of TG on gene expression are ASGPR and flotillin. An antibody against ASGPR can abrogate the inhibitory effect of TG on TTF1 and TPO promoter activity in a luciferase reporter assay in FRTL-5 cells (68). The negative feedback effects of TG on TG, NIS, TPO, DUOX2, DUOXA2, TTF1, TTF2, and PAX8 expression were also abolished by siRNAs that specifically knocked down flotillin 1 or flotillin 2 (90). How the TG signal mediated by ASGPR and flotillin is transduced to regulate the downstream genes is not clear. One candidate effector is nuclear factor 1 (NF1). Two NF1 elements were found between -264 and -153 bp in the TTF1 promoter; TG inhibits TTF1 gene expression by decreasing NF1 mRNA and protein expression and the binding activity of NF1 (259). Inhibition of TTFs including TTF1 in turn leads to suppression of TG, TPO, and NIS (260).

### Follicle heterogeneity, cycle, and feedback oscillation

While the TG-mediated feedback has been considered as an important intrafollicular mechanism for thyroid homeostasis, it is also hypothesized to act as an oscillator that drives cyclic follicular function and renders follicle heterogeneity (247, 248, 257). Follicles in a thyroid gland are highly heterogeneous in morphology and functional states (261, 262). The follicular size, TG content and colloid density of individual follicles vary greatly. ‘Active’ follicles comprise tall columnar epithelia and ‘inactive’ follicles comprise low cuboidal or squamous epithelia (263, 264). The cellular uptake and distribution of iodide also vary widely (265–267).

The heterogeneity may result from genetically different subpopulations of thyrocytes and variations in TSHR expression and downstream pathways (258, 268, 269). The follicle heterogeneity may also result from the operation of the negative feedback of TG autoregulation, which may cause cyclic changes in each follicle’s function, termed as thyroid follicular cycle (257, 270). Negative feedback is well-known for its capability of generating sustained oscillation under certain parameter conditions (271). When the colloidal iodinated TG concentration is low, the genes involved in TH synthesis, TG, NIS, TPO, DUOX2, DUOXA2, are not suppressed by TG and thus highly expressed while those genes undertaking TG degradation and TH release such as cathepsin H and MCT8 are lowly expressed. As a result, more iodinated TG is synthesized and stored in the lumen. When the TG concentration reaches a high level, it begins to suppress TH synthesis genes thus downregulating TG synthesis and iodination, and activate genes responsible for TG degradation. As a result, colloidal TG begins to be depleted, and the cyclic pattern repeats. Cycling among follicles can be asynchronous, which leads to follicle heterogeneity. Lastly, given the multiple positive autoregulation loops existing for TTFs (**Figure 2**), the possibility that the heterogeneity of thyroid follicles arises from stochastic bistable switching of the positive feedback loops between active and inactive states cannot be ruled out (272).

## Perspectives and conclusions

Homeostatic regulation of THs involves systemic interactions between multiple organs and tissues. As an integral part of this complex process, the thyroid receives physiological, dietary and environmental signals to adjust TH output on demand. These endogenous or exogenous signals impinge on the intrathyroidal molecular network comprising signal transduction pathways, TH-synthesizing and secreting pathways, and circuits regulating gene and protein expression and activities. Dysregulation of this network, either internally or externally by exogenous disruptors, can lead to pathological thyroid conditions, including goiter, hypothyroidism, hyperthyroidism, and thyroid cancers.

**Figure 6:**
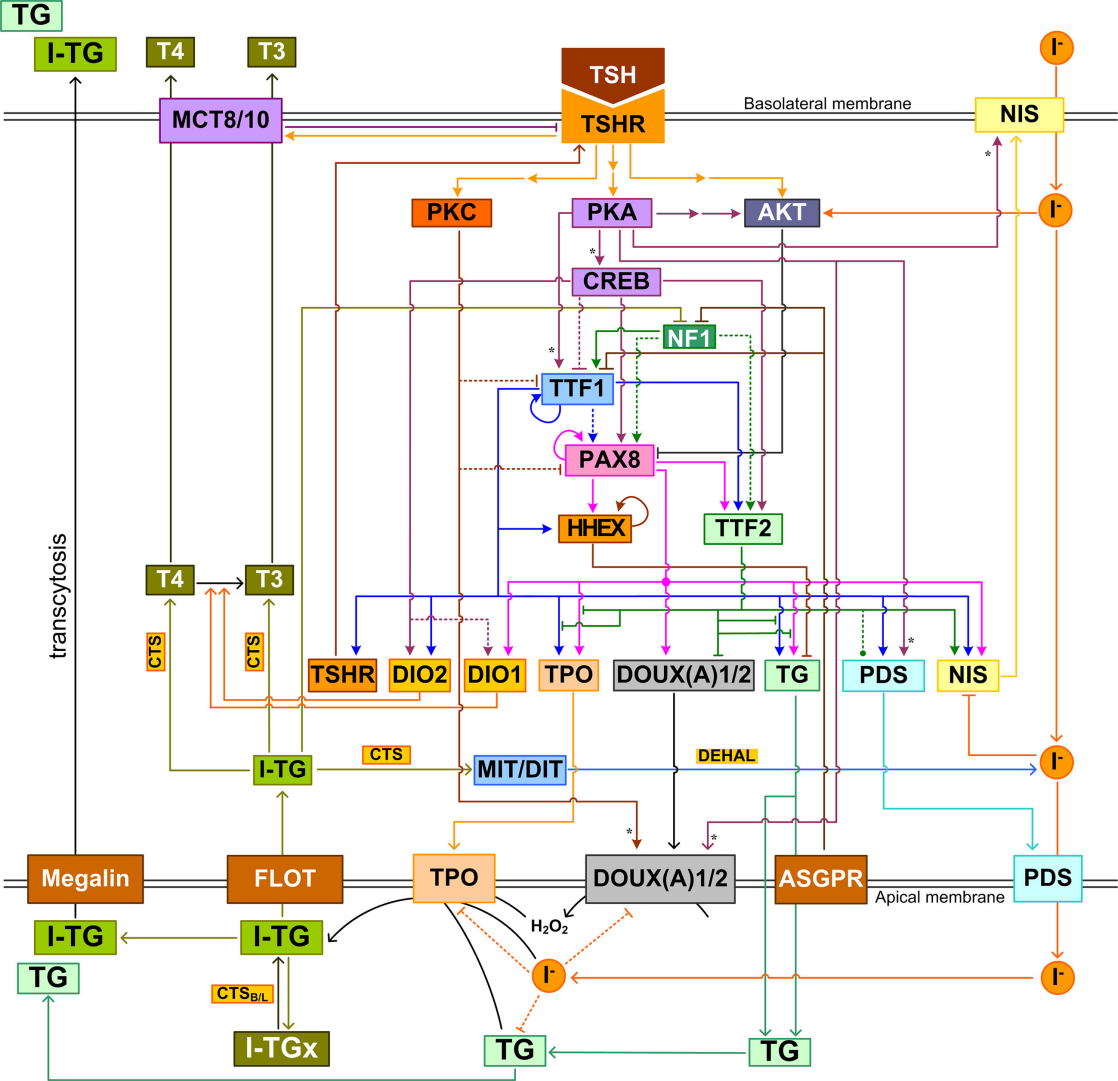
Integrated intrathyroidal feedback and feedforward regulatory network of TH synthesis and secretion. This schematic illustration shows the major signal transduction, transcriptional and posttranscriptional regulations that are composite across species. I-TGx: Cross-linked I-TG. For ease of tracking, arrow lines follow the same color as the molecule block from which the lines originate. Asterisk: phosphorylation.

As in many other intracellular systems, the structure of the intrathyroidal network is not linear and unidirectional. It involves multiple feedforward and feedback circuitries that are closely intertwined (**Figure 6**). The nonlinear properties well-known for feedforward and feedback regulations suggest that the dynamic and stimulus-response behaviors of the intrathyroidal network are not trivial, be the input signal endogenous hormones, nutrients, drugs, or EDCs. For instance, the multiple incoherent feedforward motifs formed by the signal transduction pathways, TTFs and thyroid functional genes may be responsible for the biphasic response of the thyroid to TSH. The incoherent feedforward motif driven by iodide is responsible for the TH synthesis at normal conditions and the Wolff-Chaikoff inhibition under high iodine load. Escaping from the inhibition involves a negative feedback circuit whose function seems to maintain intracellular iodide homeostasis. A potential oscillatory behavior resulting from TG-mediated negative feedback regulation may lead to cyclic follicular function and underpin the observed follicular heterogeneity (257).

More complex functions and behaviors may be expected from this network. Depending on the specific molecular target, i.e., the node in the network, the quantitative consequence of perturbation can be difficult to predict. A better understanding of complex pathways as such requires formulation of mathematical models to simulate the network as nonlinear dynamical systems (49). Such systems biology models can incorporate genetic, epigenetic, environmental and nutritional status to better predict responses of individuals. While there are many models focusing on various aspects of the HPT axis (180, 273–275), models that specifically simulate the molecular regulations in thyroid follicles are still uncommon. Degon et al. constructed a complex model describing the intrafollicular iodine turnover in humans, including uptake, apical transport, organification, endocytosis, recycling, secretion of iodide, upregulation of TPO, TG, NIS and other proteins by TSH, feedback inhibition of H_2_O_2_ production and NIS by iodide, and of TG, NIS, and TPO by TG (276). The model was used to understand and predict the condition of dietary iodine deficiency and Wolff-Chaikoff phenomena. More recently, Cohen et al. has used a bioinformatic approach to map out a regulatory network underpinning the Wolff-Chaikoff phenomena, with the goal of predicting the protective effect of repetitive dosing of iodide as a prophylactic measure (277). The intrathyroidal T4-to-T3 conversion driven by TSH-stimulated DIO2 induction was explored for its role in circadian secretion of T3 (180). Constructing more complex models based on the known intrathyroidal pathways will help identify novel molecular targets for therapeutic compounds that can regulate TH synthesis and secretion with high precision. In addition, such models can be extended to quantitative adverse outcome pathway (qAOP) models to predict dose-response relationship for health risk assessment of environmental EDCs (278–281).

In conclusion, by decomposing the complex molecular network in thyroid follicles into more tractable network motifs, such as feedforward and feedback, and re-assembling them into an organized circuitry structure, we provided a systems-biology perspective on this molecular network that regulates TH synthesis and secretion. This new perspective will help to achieve a better understanding of the network dynamics, and gain novel insights into the regulatory functions, which may provide important clues for developing future thyroid therapeutics and assessing the health impacts of environmental thyroid disruptors.

## Author contributions

QZ and LJ conceived and planned the content and structure of the review, LJ wrote the initial draft of the manuscript, and QZ and LJ critically reviewed and revised the manuscript. All authors contributed to the article and approved the submitted version.

## Funding

This research was supported in part by NIEHS Superfund Research grant: P42ES04911, NIEHS HERCULES grant P30ES019776, and the State Scholarship Fund of China Scholarship Council: 201908110055 (LJ).

## Conflict of interest

The authors declare that the research was conducted in the absence of any commercial or financial relationships that could be construed as a potential conflict of interest.

## Publisher’s note

All claims expressed in this article are solely those of the authors and do not necessarily represent those of their affiliated organizations, or those of the publisher, the editors and the reviewers. Any product that may be evaluated in this article, or claim that may be made by its manufacturer, is not guaranteed or endorsed by the publisher.

## References

[1] Jabbar A Pingitore A Pearce SH Zaman A Iervasi G Razvi S. Thyroid hormones and cardiovascular disease. Nat Rev Cardiol (2017) 14(1):39–55. doi: 10.1038/nrcardio.2016.174 27811932

[2] Yavuz S Salgado Nunez Del Prado S Celi FS. Thyroid hormone action and energy expenditure. J Endocr Soc (2019) 3(7):1345–56. doi: 10.1210/js.2018-00423 PMC6608565 31286098

[3] Silva JF Ocarino NM Serakides R. Thyroid hormones and female reproduction. Biol Reprod (2018) 99(5):907–21. doi: 10.1093/biolre/ioy115 29767691

[4] Combs CE Nicholls JJ Duncan Bassett JH Williams GR. Thyroid hormones and bone development. Minerva Endocrinol (2011) 36(1):71–85. 21460788

[5] Prezioso G Giannini C Chiarelli F. Effect of thyroid hormones on neurons and neurodevelopment. Horm Res Paediatr (2018) 90(2):73–81. doi: 10.1159/000492129 30157487

[6] Silva JE Larsen PR. Pituitary nuclear 3,5,3’-triiodothyronine and thyrotropin secretion: an explanation for the effect of thyroxine. Science (1977) 198(4317):617–20. doi: 10.1126/science.199941 199941

[7] Fonseca TL Correa-Medina M Campos MP Wittmann G Werneck-de-Castro JP Arrojo e Drigo R. Coordination of hypothalamic and pituitary T3 production regulates TSH expression. J Clin Invest (2013) 123(4):1492–500. doi: 10.1172/JCI61231 PMC3613903 23524969

[8] van der Spek AH Fliers E Boelen A. The classic pathways of thyroid hormone metabolism. Mol Cell Endocrinol (2017) 458:29–38. doi: 10.1016/j.mce.2017.01.025 28109953

[9] Luongo C Dentice M Salvatore D. Deiodinases and their intricate role in thyroid hormone homeostasis. Nat Rev Endocrinol (2019) 15(8):479–88. doi: 10.1038/s41574-019-0218-2 31160732

[10] Cavalieri RR Searle GL. The kinetics of distribution between plasma and liver of 131-i-labeled l-thyroxine in man: observations of subjects with normal and decreased serum thyroxine-binding globulin. J Clin Invest (1966) 45(6):939–49. doi: 10.1172/JCI105409 PMC292773 4958064

[11] Nicoloff JT Low JC Dussault JH Fisher DA. Simultaneous measurement of thyroxine and triiodothyronine peripheral turnover kinetics in man. J Clin Invest (1972) 51(3):473–83. doi: 10.1172/JCI106835 PMC302152 4110897

[12] Jonklaas J Burman KD Wang H Latham KR. Single-dose T3 administration: kinetics and effects on biochemical and physiological parameters. Ther Drug Monit. (2015) 37(1):110–8. doi: 10.1097/FTD.0000000000000113 PMC5167556 24977379

[13] Hillier AP. The rate of triiodothyronine dissociation from binding sites in human plasma. Acta Endocrinol (Copenh) (1975) 80(1):49–57. doi: 10.1530/acta.0.0800049 808066

[14] Mendel CM Miller MB Siiteri PK Murai JT. Rates of dissociation of steroid and thyroid hormones from human serum albumin. J Steroid Biochem Mol Biol (1990) 37(2):245–50. doi: 10.1016/0960-0760(90)90333-G 2268555

[15] Duntas LH. NEW INSIGHTS INTO THE HYPOTHALAMIC-PITUITARY-THYROID AXIS. Acta Endocrinol (Buchar) (2016) 12(2):125–9. doi: 10.4183/aeb.2016.125 PMC6535279 31149076

[16] Fekete C Lechan RM. Central regulation of hypothalamic-pituitary-thyroid axis under physiological and pathophysiological conditions. Endocr Rev (2014) 35(2):159–94. doi: 10.1210/er.2013-1087 PMC3963261 24423980

[17] Abel ED Ahima RS Boers ME Elmquist JK Wondisford FE. Critical role for thyroid hormone receptor beta2 in the regulation of paraventricular thyrotropin-releasing hormone neurons. J Clin Invest (2001) 107(8):1017–23. doi: 10.1172/JCI10858 PMC199552 11306605

[18] Hirahara N Nakamura HM Sasaki S Matsushita A Ohba K Kuroda G. Liganded T3 receptor β2 inhibits the positive feedback autoregulation of the gene for GATA2, a transcription factor critical for thyrotropin production. PLoS One (2020) 15(1):e0227646. doi: 10.1371/journal.pone.0227646 31940421 PMC6961892

[19] Marsili A Sanchez E Singru P Harney JW Zavacki AM Lechan RM. Thyroxine-induced expression of pyroglutamyl peptidase II and inhibition of TSH release precedes suppression of TRH mRNA and requires type 2 deiodinase. J Endocrinol (2011) 211(1):73–8. doi: 10.1530/JOE-11-0248 PMC3558748 21788297

[20] Jain R. Thyroid profile of the reference united states population: Data from NHANES 2007-2012. Int Arch Endocrinol Clin Res (2015) 1(1):1–8. doi: 10.23937/2572-407X.1510004

[21] Welsh KJ Soldin SJ. Diagnosis of endocrine disease: How reliable are free thyroid and total T3 hormone assays? Eur J Endocrinol (2016) 175(6):R255–r263. doi: 10.1530/EJE-16-0193 PMC5113291 27737898

[22] Mendel CM. The free hormone hypothesis: a physiologically based mathematical model. Endocr Rev (1989) 10(3):232–74. doi: 10.1210/edrv-10-3-232 2673754

[23] McLean TR Rank MM Smooker PM Richardson SJ. Evolution of thyroid hormone distributor proteins. Mol Cell Endocrinol (2017) 459:43–52. doi: 10.1016/j.mce.2017.02.038 28249735

[24] Refetoff S. Thyroid hormone serum transport proteins. In: Feingold KR Anawalt B Boyce A, editors. Endotext. South Dartmouth (MA: MDText.com, Inc. Copyright © 2000-2021 (2000). 25905421

[25] Benvenga S Alesci S Trimarchi F. High-density lipoprotein-facilitated entry of thyroid hormones into cells: a mechanism different from the low-density lipoprotein-facilitated entry. Thyroid (2002) 12(7):547–56. doi: 10.1089/105072502320288384 12193297

[26] Schussler GC. The thyroxine-binding proteins. Thyroid (2000) 10(2):141–9. doi: 10.1089/thy.2000.10.141 10718550

[27] Richardson SJ. Cell and molecular biology of transthyretin and thyroid hormones. Int Rev Cytol. (2007) 258:137–93. doi: 10.1016/S0074-7696(07)58003-4 17338921

[28] Pappa T Ferrara AM Refetoff S. Inherited defects of thyroxine-binding proteins. Best Pract Res Clin Endocrinol Metab (2015) 29(5):735–47. doi: 10.1016/j.beem.2015.09.002 PMC4632647 26522458

[29] Peeters RP Visser TJ. Metabolism of thyroid hormone. In: Feingold KR Anawalt B Boyce A, editors. Endotext. South Dartmouth (MA: MDText.com, Inc. Copyright © 2000-2021 (2000).

[30] Kim SW Harney JW Larsen PR. Studies of the hormonal regulation of type 2 5’-iodothyronine deiodinase messenger ribonucleic acid in pituitary tumor cells using semiquantitative reverse transcription-polymerase chain reaction. Endocrinology (1998) 139(12):4895–905. doi: 10.1210/endo.139.12.6334 9832426

[31] Hosoi Y Murakami M Mizuma H Ogiwara T Imamura M Mori M. Expression and regulation of type II iodothyronine deiodinase in cultured human skeletal muscle cells. J Clin Endocrinol Metab (1999) 84(9):3293–300. doi: 10.1210/jc.84.9.3293 10487701

[32] Bianco AC Dumitrescu A Gereben B Ribeiro MO Fonseca TL Fernandes GW. Paradigms of dynamic control of thyroid hormone signaling. Endocr Rev (2019) 40(4):1000–47. doi: 10.1210/er.2018-00275 PMC6596318 31033998

[33] Rastogi L Godbole MM Sinha RA Pradhan S. Reverse triiodothyronine (rT3) attenuates ischemia-reperfusion injury. Biochem Biophys Res Commun (2018) 506(3):597–603. doi: 10.1016/j.bbrc.2018.10.031 PMC7212030 30366665

[34] Toyoda N Zavacki AM Maia AL Harney JW Larsen PR. A novel retinoid X receptor-independent thyroid hormone response element is present in the human type 1 deiodinase gene. Mol Cell Biol (1995) 15(9):5100–12. doi: 10.1128/MCB.15.9.5100 PMC230757 7651427

[35] Zhang CY Kim S Harney JW Larsen PR. Further characterization of thyroid hormone response elements in the human type 1 iodothyronine deiodinase gene. Endocrinology (1998) 139(3):1156–63. doi: 10.1210/endo.139.3.5849 9492050

[36] Zavacki AM Ying H Christoffolete MA Aerts G So E Harney JW. Type 1 iodothyronine deiodinase is a sensitive marker of peripheral thyroid status in the mouse. Endocrinology (2005) 146(3):1568–75. doi: 10.1210/en.2004-1392 15591136

[37] Steinsapir J Harney J Larsen PR. Type 2 iodothyronine deiodinase in rat pituitary tumor cells is inactivated in proteasomes. J Clin Invest (1998) 102(11):1895–9. doi: 10.1172/JCI4672 PMC509140 9835613

[38] Gereben B Goncalves C Harney JW Larsen PR Bianco AC. Selective proteolysis of human type 2 deiodinase: a novel ubiquitin-proteasomal mediated mechanism for regulation of hormone activation. Mol Endocrinol (2000) 14(11):1697–708. doi: 10.1210/mend.14.11.0558 11075806

[39] Steinsapir J Bianco AC Buettner C Harney J Larsen PR. Substrate-induced down-regulation of human type 2 deiodinase (hD2) is mediated through proteasomal degradation and requires interaction with the enzyme’s active center. Endocrinology (2000) 141(3):1127–35. doi: 10.1210/endo.141.3.7355 10698189

[40] Sagar GD Gereben B Callebaut I Mornon JP Zeöld A da Silva WS. Ubiquitination-induced conformational change within the deiodinase dimer is a switch regulating enzyme activity. Mol Cell Biol (2007) 27(13):4774–83. doi: 10.1128/MCB.00283-07 PMC1951476 17452445

[41] Kaplan MM Yaskoski KA. Phenolic and tyrosyl ring deiodination of iodothyronines in rat brain homogenates. J Clin Invest (1980) 66(3):551–62. doi: 10.1172/JCI109887 PMC371684 7400328

[42] Esfandiari A Courtin F Lennon AM Gavaret JM Pierre M. Induction of type III deiodinase activity in astroglial cells by thyroid hormones. Endocrinology (1992) 131(4):1682–8. doi: 10.1210/endo.131.4.1396314 1396314

[43] Becker KB Stephens KC Davey JC Schneider MJ Galton VA. The type 2 and type 3 iodothyronine deiodinases play important roles in coordinating development in rana catesbeiana tadpoles. Endocrinology (1997) 138(7):2989–97. doi: 10.1210/endo.138.7.5272 9202244

[44] Masmoudi T Planells R Mounié J Artur Y Magdalou J Goudonnet H. Opposite regulation of bilirubin and 4-nitrophenol UDP-glucuronosyltransferase mRNA levels by 3,3’,5 triiodo-l-thyronine in rat liver. FEBS Lett (1996) 379(2):181–5. doi: 10.1016/0014-5793(95)01507-8 8635588

[45] Masmoudi T Mounié J Artur Y Magdalou J Goudonnet H. Comparative quantification of two hepatic UDP-glucuronosyltransferase bilirubin isoforms mRNAs in various thyroid states in rat. Biochem Pharmacol (1997) 53(7):1013–7. doi: 10.1016/S0006-2952(96)00886-6 9174115

[46] Li YQ Prentice DA Howard ML Mashford ML Desmond PV. The effect of hormones on the expression of five isoforms of UDP-glucuronosyltransferase in primary cultures of rat hepatocytes. Pharm Res (1999) 16(2):191–7. doi: 10.1023/A:1018812021549 10100302

[47] Masmoudi T Mounié J Artur Y Magdalou J Goudonnet H. Comparative quantification of two hepatic UDP-glucuronosyltransferase bilirubin isoforms mRNAs in various thyroid states in rat. Biochem Pharmacol (1997) 53(7):1013–7. doi: 10.1016/S0006-2952(96)00886-6 9174115

[48] Alon U. Network motifs: theory and experimental approaches. Nat Rev Genet (2007) 8(6):450–61. doi: 10.1038/nrg2102 17510665

[49] El-Samad H. Biological feedback control–respect the loops. Cell Syst (2021) 12(6):477–87. doi: 10.1016/j.cels.2021.05.004 34139160

[50] Fujita H. Functional morphology of the thyroid. Int Rev Cytol. (1988) 113:145–85. doi: 10.1016/S0074-7696(08)60848-7 3068180

[51] Ravera S Reyna-Neyra A Ferrandino G Amzel LM Carrasco N. The Sodium/Iodide symporter (NIS): Molecular physiology and preclinical and clinical applications. Annu Rev Physiol (2017) 79:261–89. doi: 10.1146/annurev-physiol-022516-034125 PMC5739519 28192058

[52] Rousset B Dupuy C Miot F Dumont J. Thyroid hormone synthesis and secretion. In: Feingold KR Anawalt B Boyce A Chrousos G Herder WW Dungan K, editors. Endotext. South Dartmouth (MA: MDText.com, Inc.Copyright © 2000-2021 (2000).

[53] Gillam MP Sidhaye AR Lee EJ Rutishauser J Stephan CW Kopp P. Functional characterization of pendrin in a polarized cell system. evidence for pendrin-mediated apical iodide efflux. J Biol Chem (2004) 279(13):13004–10. doi: 10.1074/jbc.M313648200 14715652

[54] Twyffels L Strickaert A Virreira M Massart C Van Sande J Wauquier C. Anoctamin-1/TMEM16A is the major apical iodide channel of the thyrocyte. Am J Physiol Cell Physiol (2014) 307(12):C1102–1112. doi: 10.1152/ajpcell.00126.2014 25298423

[55] Yoshida A Taniguchi S Hisatome I Royaux IE Green ED Kohn LD. Pendrin is an iodide-specific apical porter responsible for iodide efflux from thyroid cells. J Clin Endocrinol Metab (2002) 87(7):3356–61. doi: 10.1210/jcem.87.7.8679 12107249

[56] Ishii J Suzuki A Kimura T Tateyama M Tanaka T Yazawa T. Congenital goitrous hypothyroidism is caused by dysfunction of the iodide transporter SLC26A7. Commun Biol (2019) 2:270. doi: 10.1038/s42003-019-0503-6 31372509 PMC6656751

[57] Poncelet L Dumont JE Miot F De Deken X. The dual oxidase Duox2 stabilized with DuoxA2 in an enzymatic complex at the surface of the cell produces extracellular H(2)O(2) able to induce DNA damage in an inducible cellular model. Exp Cell Res (2019) 384(1):111620. doi: 10.1016/j.yexcr.2019.111620 31513783

[58] Sarr D Tóth E Gingerich A Rada B. Antimicrobial actions of dual oxidases and lactoperoxidase. J Microbiol (2018) 56(6):373–86. doi: 10.1007/s12275-018-7545-1 PMC7336354 29858825

[59] Rigutto S Hoste C Grasberger H Milenkovic M Communi D Dumont JE. Activation of dual oxidases Duox1 and Duox2: differential regulation mediated by camp-dependent protein kinase and protein kinase c-dependent phosphorylation. J Biol Chem (2009) 284(11):6725–34. doi: 10.1074/jbc.M806893200 PMC2652333 19144650

[60] Carvalho DP Dupuy C. Thyroid hormone biosynthesis and release. Mol Cell Endocrinol (2017) 458:6–15. doi: 10.1016/j.mce.2017.01.038 28153798

[61] Senou M Costa MJ Massart C Thimmesch M Khalifa C Poncin S. Role of caveolin-1 in thyroid phenotype, cell homeostasis, and hormone synthesis: *in vivo* study of caveolin-1 knockout mice. Am J Physiol Endocrinol Metab (2009) 297(2):E438–451. doi: 10.1152/ajpendo.90784.2008 19435853

[62] Senou M Khalifa C Thimmesch M Jouret F Devuyst O Col V. A coherent organization of differentiation proteins is required to maintain an appropriate thyroid function in the pendred thyroid. J Clin Endocrinol Metab (2010) 95(8):4021–30. doi: 10.1210/jc.2010-0228 20501687

[63] Godlewska M Banga PJ. Thyroid peroxidase as a dual active site enzyme: Focus on biosynthesis, hormonogenesis and thyroid disorders of autoimmunity and cancer. Biochimie (2019) 160:34–45. doi: 10.1016/j.biochi.2019.02.003 30742860

[64] Fortunato RS Lima de Souza EC Ameziane-el Hassani R Boufraqech M Weyemi U Talbot M. Functional consequences of dual oxidase-thyroperoxidase interaction at the plasma membrane”. J Clin Endocrinol Metab (2010) 95(12):5403–11. doi: 10.1210/jc.2010-1085 20826581

[65] Song Y Ruf J Lothaire P Dequanter D Andry G Willemse E. Association of duoxes with thyroid peroxidase and its regulation in thyrocytes. J Clin Endocrinol Metab (2010) 95(1):375–82. doi: 10.1210/jc.2009-1727 19952225

[66] Citterio CE Targovnik HM Arvan P. The role of thyroglobulin in thyroid hormonogenesis. Nat Rev Endocrinol (2019) 15(6):323–38. doi: 10.1038/s41574-019-0184-8 30886364

[67] Marinò M McCluskey RT. Role of thyroglobulin endocytic pathways in the control of thyroid hormone release. Am J Physiol Cell Physiol (2000) 279(5):C1295–1306. doi: 10.1152/ajpcell.2000.279.5.C1295 11029276

[68] Ulianich L Suzuki K Mori A Nakazato M Pietrarelli M Goldsmith P. Follicular thyroglobulin (TG) suppression of thyroid-restricted genes involves the apical membrane asialoglycoprotein receptor and TG phosphorylation. J Biol Chem (1999) 274(35):25099–107. doi: 10.1074/jbc.274.35.25099 10455190

[69] Lamas L Anderson PC Fox JW Dunn JT . Consensus sequences for early iodination and hormonogenesis in human thyroglobulin. J Biol Chem. (1989) 264(23):13541–5. doi: 10.1016/S0021-9258(18)80031-X 2760035

[70] Dunn AD Corsi CM Myers HE Dunn JT. Tyrosine 130 is an important outer ring donor for thyroxine formation in thyroglobulin. J Biol Chem (1998) 273(39):25223–9. doi: 10.1074/jbc.273.39.25223 9737985

[71] Coscia F Taler-Verčič A Chang VT Sinn L O’Reilly FJ Izoré T. The structure of human thyroglobulin. Nature (2020) 578(7796):627–30. doi: 10.1038/s41586-020-1995-4 PMC7170718 32025030

[72] Citterio CE Rivolta CM Targovnik HM. Structure and genetic variants of thyroglobulin: Pathophysiological implications. Mol Cell Endocrinol (2021) 528:111227. doi: 10.1016/j.mce.2021.111227 33689781

[73] Gavaret JM Cahnmann HJ Nunez J. Thyroid hormone synthesis in thyroglobulin. the mechanism of the coupling reaction. J Biol Chem (1981) 256(17):9167–73. doi: 10.1016/S0021-9258(19)52523-6 7021557

[74] Lerman J. Iodine components of the blood. Circulating thyroglobulin in normal persons and in persons with thyroid disease. J Clin Invest (1940) 19(4):555–60. doi: 10.1172/JCI101158 PMC434990 16694772

[75] Tong W Taurog A Chaikoff IL. Non-thyroglobulin iodine of the thyroid gland And diiodotyrosine. J Biol Chem (1951) 191(2):665–75. doi: 10.1016/S0021-9258(18)55971-8 14861212

[76] Herzog V Berndorfer U Saber Y. Isolation of insoluble secretory product from bovine thyroid: extracellular storage of thyroglobulin in covalently cross-linked form. J Cell Biol (1992) 118(5):1071–83. doi: 10.1083/jcb.118.5.1071 PMC2289578 1512290

[77] Brix K Szumska J Weber J Qatato M Venugopalan V Al-Hashimi A. Auto-regulation of the thyroid gland beyond classical pathways. Exp Clin Endocrinol Diabetes (2020) 128(6-07):437–45. doi: 10.1055/a-1080-2969 32074633

[78] Hayden LJ Shagrin JM Young JA. Micropuncture investigation of the anion content of colloid from single rat thyroid follicles. a micromethod for the simultaneous determination of iodide and chloride in nanomole quantities. Pflugers Arch (1970) 321(2):173–86. doi: 10.1007/BF00586371 5529539

[79] Smeds S. A microgel electrophoretic analysis of the colloid proteins in single rat thyroid follicles. II. the protein concentration of the colloid single rat thyroid follicles. Endocrinology (1972) 91(5):1300–6. doi: 10.1210/endo-91-5-1288 5072808

[80] Berndorfer U Wilms H Herzog V. Multimerization of thyroglobulin (TG) during extracellular storage: isolation of highly cross-linked TG from human thyroids. J Clin Endocrinol Metab (1996) 81(5):1918–26. doi: 10.1210/jcem.81.5.8626858 8626858

[81] Loewenstein JE Wollman SH. Diffusion of thyroglobulin in the lumen of rat thyroid follicle. Endocrinology (1967) 81(5):1086–90. doi: 10.1210/endo-81-5-1086 6052937

[82] Haeberli A Engler H von Grünigen C Kohler H Studer H. Low molecular weight intracellular iodocompounds with long intrathyroidal half-life: remnants of thyroglobulin hydrolysis? Acta Endocrinol (Copenh) (1979) 92(1):105–18. doi: 10.1530/acta.0.0920105 494983

[83] Bagchi N Brown TR Shivers B Mack RE. Role of the heterogeneity of thyroglobulin in the secretion of thyroid hormone in mice. J Endocrinol (1980) 86(3):413–8. doi: 10.1677/joe.0.0860413 7430900

[84] Schneider PB. Thyroidal iodine heterogeneity: “Last come, first served. Syst. Of iodine turnover” Endocrinol (1964) 74:973–80. doi: 10.1210/endo-74-6-973 14190639

[85] Brix K Linke M Tepel C Herzog V. Cysteine proteinases mediate extracellular prohormone processing in the thyroid. Biol Chem (2001) 382(5):717–25. doi: 10.1515/bchm.2001.382.5.717 11517924

[86] Brix K Lemansky P Herzog V. Evidence for extracellularly acting cathepsins mediating thyroid hormone liberation in thyroid epithelial cells. Endocrinology (1996) 137(5):1963–74. doi: 10.1210/endo.137.5.8612537 8612537

[87] Lemansky P Brix K Herzog V. Iodination of mature cathepsin d in thyrocytes as an indicator for its transport to the cell surface. Eur J Cell Biol (1998) 76(1):53–62. doi: 10.1016/S0171-9335(98)80017-4 9650783

[88] Tepel C Brömme D Herzog V Brix K. Cathepsin K in thyroid epithelial cells: sequence, localization and possible function in extracellular proteolysis of thyroglobulin. J Cell Sci (2000) 113 Pt 24:4487–98. doi: 10.1242/jcs.113.24.4487 11082042

[89] Friedrichs B Tepel C Reinheckel T Deussing J von Figura K Herzog V. “Thyroid functions of mouse cathepsins b, K and l. J Clin Invest (2003) 111(11):1733–45. doi: 10.1172/JCI15990 PMC156100 12782676

[90] Luo Y Akama T Okayama A Yoshihara A Sue M Oda K. A novel role for flotillin-containing lipid rafts in negative-feedback regulation of thyroid-specific gene expression by thyroglobulin. Thyroid (2016) 26(11):1630–9. doi: 10.1089/thy.2016.0187 27676653

[91] Marinò M Zheng G Chiovato L Pinchera A Brown D Andrews D. Role of megalin (gp330) in transcytosis of thyroglobulin by thyroid cells. a novel function in the control of thyroid hormone release. J Biol Chem (2000) 275(10):7125–37. doi: 10.1074/jbc.275.10.7125 10702280

[92] Consiglio E Salvatore G Rall JE Kohn LD. Thyroglobulin interactions with thyroid plasma membranes. the existence of specific receptors and their potential role. J Biol Chem (1979) 254(12):5065–76. doi: 10.1016/S0021-9258(18)50561-5 221458

[93] Miquelis R Courageot J Jacq A Blanck O Perrin C Bastiani P. Intracellular routing of GLcNAc-bearing molecules in thyrocytes: selective recycling through the golgi apparatus. J Cell Biol (1993) 123(6 Pt 2):1695–706. doi: 10.1083/jcb.123.6.1695 PMC2290866 7506265

[94] Bastiani P Papandreou MJ Blanck O Fenouillet E Thibault V Miquelis R. On the relationship between completion of n-acetyllactosamine oligosaccharide units and iodine content of thyroglobulin: a reinvestigation. Endocrinology (1995) 136(10):4204–9. doi: 10.1210/endo.136.10.7664637 7664637

[95] Dunn AD Crutchfield HE Dunn JT. Thyroglobulin processing by thyroidal proteases. major sites of cleavage by cathepsins b, d and l. J Biol Chem (1991) 266(30):20198–204. doi: 10.1016/S0021-9258(18)54909-7 1939080

[96] Dunn AD Crutchfield HE Dunn JT. Proteolytic processing of thyroglobulin by extracts of thyroid lysosomes. Endocrinology (1991) 128(6):3073–80. doi: 10.1210/endo-128-6-3073 1903699

[97] Rokita SE Adler JM McTamney PM Watson JA Jr. Efficient use and recycling of the micronutrient iodide in mammals. Biochimie (2010) 92(9):1227–35. doi: 10.1016/j.biochi.2010.02.013 PMC2888766 20167242

[98] Gnidehou S Caillou B Talbot M Ohayon R Kaniewski J Noël-Hudson MS. Iodotyrosine dehalogenase 1 (DEHAL1) is a transmembrane protein involved in the recycling of iodide close to the thyroglobulin iodination site. FASEB J (2004) 18(13):1574–6. doi: 10.1096/fj.04-2023fje 15289438

[99] Solís SJ Villalobos P Orozco A Valverde RC. Comparative kinetic characterization of rat thyroid iodotyrosine dehalogenase and iodothyronine deiodinase type 1. J Endocrinol (2004) 181(3):385–92. doi: 10.1677/joe.0.1810385 15171686

[100] Köhrle J. Local activation and inactivation of thyroid hormones: the deiodinase family. Mol Cell Endocrinol (1999) 151(1-2):103–19. doi: 10.1016/S0303-7207(99)00040-4 10411325

[101] Trajkovic-Arsic M Müller J Darras VM Groba C Lee S Weih D. Impact of monocarboxylate transporter-8 deficiency on the hypothalamus-pituitary-thyroid axis in mice. Endocrinology (2010) 151(10):5053–62. doi: 10.1210/en.2010-0593 20702572

[102] de Souza EC Dias GR Cardoso RC Lima LP Fortunato RS Visser TJ. MCT8 is downregulated by short time iodine overload in the thyroid gland of rats. Horm Metab Res (2015) 47(12):910–5. doi: 10.1055/s-0035-1550008 26021458

[103] Friesema EC Jansen J Jachtenberg JW Visser WE Kester MH Visser TJ. Effective cellular uptake and efflux of thyroid hormone by human monocarboxylate transporter 10. Mol Endocrinol (2008) 22(6):1357–69. doi: 10.1210/me.2007-0112 PMC5419535 18337592

[104] Johannes J Braun D Kinne A Rathmann D Köhrle J Schweizer U. Few amino acid exchanges expand the substrate spectrum of monocarboxylate transporter 10. Mol Endocrinol (2016) 30(7):796–808. doi: 10.1210/me.2016-1037 PMC5426580 27244477

[105] Müller J Mayerl S Visser TJ Darras VM Boelen A Frappart L. Tissue-specific alterations in thyroid hormone homeostasis in combined Mct10 and Mct8 deficiency. Endocrinology (2014) 155(1):315–25. doi: 10.1210/en.2013-1800 24248460

[106] Groeneweg S van Geest FS Peeters RP Heuer H Visser WE. Thyroid hormone transporters. Endocr Rev (2020) 41(2):146–201. doi: 10.1210/endrev/bnz008 31754699

[107] Rapoport B Chazenbalk GD Jaume JC McLachlan SM. The thyrotropin (TSH) receptor: interaction with TSH and autoantibodies. Endocr Rev (1998) 19(6):673–716. doi: 10.1210/edrv.19.6.0352 9861544

[108] Korta P Pocheć E. Glycosylation of thyroid-stimulating hormone receptor. Endokrynol. Pol (2019) 70(1):86–100. doi: 10.5603/EP.a2018.0077 30843179

[109] Rapoport B McLachlan SM. TSH receptor cleavage into subunits and shedding of the a-subunit; a molecular and clinical perspective. Endocr Rev (2016) 37(2):114–34. doi: 10.1210/er.2015-1098 PMC4823380 26799472

[110] Zoenen M Urizar E Swillens S Vassart G Costagliola S. Evidence for activity-regulated hormone-binding cooperativity across glycoprotein hormone receptor homomers. Nat Commun (2012) 3:1007. doi: 10.1038/ncomms1991 22893131

[111] Kleinau G Worth CL Kreuchwig A Biebermann H Marcinkowski P Scheerer P. Structural-functional features of the thyrotropin receptor: A class a G-Protein-Coupled receptor at work. Front Endocrinol (Lausanne) (2017) 8:86. doi: 10.3389/fendo.2017.00086 28484426 PMC5401882

[112] Zaballos MA Garcia B Santisteban P. Gbetagamma dimers released in response to thyrotropin activate phosphoinositide 3-kinase and regulate gene expression in thyroid cells. Mol Endocrinol (2008) 22(5):1183–99. doi: 10.1210/me.2007-0093 PMC5419518 18202153

[113] Laugwitz KL Allgeier A Offermanns S Spicher K Van Sande J Dumont JE. The human thyrotropin receptor: a heptahelical receptor capable of stimulating members of all four G protein families. Proc Natl Acad Sci U.S.A. (1996) 93(1):116–20. doi: 10.1073/pnas.93.1.116 PMC40189 8552586

[114] Latif R Morshed SA Ma R Tokat B Mezei M Davies TF. A gq biased small molecule active at the TSH receptor. Front Endocrinol (Lausanne) (2020) 11:372. doi: 10.3389/fendo.2020.00372 32676053 PMC7333667

[115] Shang H Zhao J Yao J Wang H Dong J Liao L. Nevirapine increases Sodium/Iodide symporter-mediated radioiodide uptake by activation of TSHR/cAMP/CREB/PAX8 signaling pathway in dedifferentiated thyroid cancer. Front Oncol (2020) 10:404. doi: 10.3389/fonc.2020.00404 32300552 PMC7145398

[116] Allgeier A Offermanns S Van Sande J Spicher K Schultz G Dumont JE. The human thyrotropin receptor activates G-proteins gs and Gq/11. J Biol Chem (1994) 269(19):13733–5. doi: 10.1016/S0021-9258(17)36705-4 8188646

[117] Laurent E Mockel J Van Sande J Graff I Dumont JE. Dual activation by thyrotropin of the phospholipase c and cyclic AMP cascades in human thyroid. Mol Cell Endocrinol (1987) 52(3):273–8. doi: 10.1016/0303-7207(87)90055-4 2820816

[118] Van Sande J Lejeune C Ludgate M Munro DS Vassart G Dumont JE. Thyroid stimulating immunoglobulins, like thyrotropin activate both the cyclic AMP and the PIP2 cascades in CHO cells expressing the TSH receptor. Mol Cell Endocrinol (1992) 88(1-3):R1–5. doi: 10.1016/0303-7207(92)90024-Z 1360926

[119] Hidaka A Minegishi T Kohn LD. Thyrotropin, like luteinizing hormone (LH) and chorionic gonadotropin (CG), increases cAMP and inositol phosphate levels in cells with recombinant human LH/CG receptor. Biochem Biophys Res Commun (1993) 196(1):187–95. doi: 10.1006/bbrc.1993.2233 8216292

[120] Allen MD Neumann S Gershengorn MC. Occupancy of both sites on the thyrotropin (TSH) receptor dimer is necessary for phosphoinositide signaling. FASEB J (2011) 25(10):3687–94. doi: 10.1096/fj.11-188961 PMC3177577 21705666

[121] Calebiro D de Filippis T Lucchi S Martinez F Porazzi P Trivellato R. Selective modulation of protein kinase a I and II reveals distinct roles in thyroid cell gene expression and growth. Mol Endocrinol (2006) 20(12):3196–211. doi: 10.1210/me.2005-0493 16887886

[122] Godbole A Lyga S Lohse MJ Calebiro D. Internalized TSH receptors en route to the TGN induce local g(s)-protein signaling and gene transcription. Nat Commun (2017) 8(1):443. doi: 10.1038/s41467-017-00357-2 28874659 PMC5585343

[123] Ribeiro-Neto F Urbani J Lemee N Lou L Altschuler DL. On the mitogenic properties of Rap1b: cAMP-induced G(1)/S entry requires activated and phosphorylated Rap1b. Proc Natl Acad Sci U.S.A. (2002) 99(8):5418–23. doi: 10.1073/pnas.082122499 PMC122784 11959997

[124] Ariga M Nedachi T Akahori M Sakamoto H Ito Y Hakuno F. Signalling pathways of insulin-like growth factor-I that are augmented by cAMP in FRTL-5 cells. Biochem J (2000) 348 Pt 2(Pt 2):409–16. doi: 10.1042/bj3480409 PMC1221080 10816436

[125] Cass LA Meinkoth JL. Ras signaling through PI3K confers hormone-independent proliferation that is compatible with differentiation. Oncogene (2000) 19(7):924–32. doi: 10.1038/sj.onc.1203393 10702801

[126] Kimura T Van Keymeulen A Golstein J Fusco A Dumont JE Roger PP. Regulation of thyroid cell proliferation by TSH and other factors: a critical evaluation of *in vitro* models. Endocr Rev (2001) 22(5):631–56. doi: 10.1210/edrv.22.5.0444 11588145

[127] Saavedra AP Tsygankova OM Prendergast GV Dworet JH Cheng G Meinkoth JL. Role of cAMP, PKA and Rap1A in thyroid follicular cell survival. Oncogene (2002) 21(5):778–88. doi: 10.1038/sj.onc.1205123 11850806

[128] Cass LA Meinkoth JL. Differential effects of cyclic adenosine 3’,5’-monophosphate on p70 ribosomal S6 kinase. Endocrinology (1998) 139(4):1991–8. doi: 10.1210/endo.139.4.5880 9528986

[129] Ginsberg J Matowe W Murray PG. Enhancement of thyrotropin-stimulated iodide organification in porcine thyroid cells after protein kinase-c inhibition. Endocrinology (1993) 132(4):1815–9. doi: 10.1210/endo.132.4.8462478 8462478

[130] Linke M Jordans S Mach L Herzog V Brix K. Thyroid stimulating hormone upregulates secretion of cathepsin b from thyroid epithelial cells. Biol Chem (2002) 383(5):773–84. doi: 10.1515/BC.2002.081 12108542

[131] Kero J Ahmed K Wettschureck N Tunaru S Wintermantel T Greiner E. Thyrocyte-specific Gq/G11 deficiency impairs thyroid function and prevents goiter development. J Clin Invest (2007) 117(9):2399–407. doi: 10.1172/JCI30380 PMC1937498 17694176

[132] Fernandez N Caloca MJ Prendergast GV Meinkoth JL Kazanietz MG. Atypical protein kinase c-zeta stimulates thyrotropin-independent proliferation in rat thyroid cells. Endocrinology (2000) 141(1):146–52. doi: 10.1210/endo.141.1.7278 10614633

[133] Sho KM Okajima F Abdul Majid M Kondo Y. Reciprocal modulation of thyrotropin actions by P1-purinergic agonists in FRTL-5 thyroid cells. inhibition of cAMP pathway and stimulation of phospholipase c-Ca2+ pathway. J Biol Chem (1991) 266(19):12180–4. doi: 10.1016/S0021-9258(18)98877-0 1648085

[134] Gallo A Benusiglio E Bonapace IM Feliciello A Cassano S Garbi C. V-ras and protein kinase c dedifferentiate thyroid cells by down-regulating nuclear cAMP-dependent protein kinase a. Genes Dev (1992) 6(9):1621–30. doi: 10.1101/gad.6.9.1621 1325391

[135] Hur EM Kim KT. G Protein-coupled receptor signalling and cross-talk: achieving rapidity and specificity. Cell Signal (2002) 14(5):397–405. doi: 10.1016/S0898-6568(01)00258-3 11882384

[136] Lesage GD Marucci L Alvaro D Glaser SS Benedetti A Marzioni M. Insulin inhibits secretin-induced ductal secretion by activation of PKC alpha and inhibition of PKA activity. Hepatology (2002) 36(3):641–51. doi: 10.1053/jhep.2002.35537 12198656

[137] Laglia G Zeiger MA Leipricht A Caturegli P Levine MA Kohn LD. Increased cyclic adenosine 3’,5’-monophosphate inhibits G protein-coupled activation of phospholipase c in rat FRTL-5 thyroid cells. Endocrinology (1996) 137(8):3170–6. doi: 10.1210/endo.137.8.8754735 8754735

[138] Boutin A Krieger CC Marcus-Samuels B Klubo-Gwiezdzinska J Neumann S Gershengorn MC. TSH receptor homodimerization in regulation of cAMP production in human thyrocytes *in vitro* . Front Endocrinol (2020) 11:276. doi: 10.3389/fendo.2020.00276 PMC7203478 32425890

[139] Neumann S Malik SS Marcus-Samuels B Eliseeva E Jang D Klubo-Gwiezdzinska J. Thyrotropin causes dose-dependent biphasic regulation of cAMP production mediated by g(s) and g(i/o) proteins. Mol Pharmacol (2020) 97(1):2–8. doi: 10.1124/mol.119.117382 PMC6864415 31704717

[140] Büch TR Biebermann H Kalwa H Pinkenburg O Hager D Barth H. G13-dependent activation of MAPK by thyrotropin. J Biol Chem (2008) 283(29):20330–41. doi: 10.1074/jbc.M800211200 18445595

[141] Fernández LP López-Márquez A Santisteban P. Thyroid transcription factors in development, differentiation and disease. Nat Rev Endocrinol (2015) 11(1):29–42. doi: 10.1038/nrendo.2014.186 25350068

[142] Suzuki K Lavaroni S Mori A Ohta M Saito J Pietrarelli M. Autoregulation of thyroid-specific gene transcription by thyroglobulin. Proc Natl Acad Sci U.S.A. (1998) 95(14):8251–6. doi: 10.1073/pnas.95.14.8251 PMC20962 9653173

[143] López-Márquez A Carrasco-López C Fernández-Méndez C Santisteban P. Unraveling the complex interplay between transcription factors and signaling molecules in thyroid differentiation and function, from embryos to adults. Front Endocrinol (Lausanne) (2021) 12:654569. doi: 10.3389/fendo.2021.654569 33959098 PMC8095082

[144] Sastre-Perona A Santisteban P. Wnt-independent role of β-catenin in thyroid cell proliferation and differentiation. Mol Endocrinol (2014) 28(5):681–95. doi: 10.1210/me.2013-1377 PMC5414850 24645679

[145] Di Palma T Nitsch R Mascia A Nitsch L Di Lauro R Zannini M. The paired domain-containing factor Pax8 and the homeodomain-containing factor TTF-1 directly interact and synergistically activate transcription. J Biol Chem (2003) 278(5):3395–402. doi: 10.1074/jbc.M205977200 12441357

[146] Puppin C D’Elia AV Pellizzari L Russo D Arturi F Presta I. Thyroid-specific transcription factors control hex promoter activity. Nucleic Acids Res (2003) 31(7):1845–52. doi: 10.1093/nar/gkg295 PMC152810 12655000

[147] Puppin C Presta I D’Elia AV Tell G Arturi F Russo D. Functional interaction among thyroid-specific transcription factors: Pax8 regulates the activity of hex promoter. Mol Cell Endocrinol (2004) 214(1-2):117–25. doi: 10.1016/j.mce.2003.10.061 15062550

[148] Cuesta I Zaret KS Santisteban P. The forkhead factor FoxE1 binds to the thyroperoxidase promoter during thyroid cell differentiation and modifies compacted chromatin structure. Mol Cell Biol (2007) 27(20):7302–14. doi: 10.1128/MCB.00758-07 PMC2168900 17709379

[149] Dame K Cincotta S Lang AH Sanghrajka RM Zhang L Choi J. Thyroid progenitors are robustly derived from embryonic stem cells through transient, developmental stage-specific overexpression of Nkx2-1. Stem Cell Rep (2017) 8(2):216–25. doi: 10.1016/j.stemcr.2016.12.024 PMC5312259 28162994

[150] D’Andrea B Iacone R Di Palma T Nitsch R Baratta MG Nitsch L. Functional inactivation of the transcription factor Pax8 through oligomerization chain reaction. Mol Endocrinol (2006) 20(8):1810–24. doi: 10.1210/me.2005-0463 16613988

[151] Christophe-Hobertus C Lefort A Libert F Christophe D. Functional inactivation of thyroid transcription factor-1 in PCCl3 thyroid cells. Mol Cell Endocrinol (2012) 358(1):36–45. doi: 10.1016/j.mce.2012.02.013 22370158

[152] Oguchi H Kimura S. Multiple transcripts encoded by the thyroid-specific enhancer-binding protein (T/EBP)/thyroid-specific transcription factor-1 (TTF-1) gene: evidence of autoregulation. Endocrinology (1998) 139(4):1999–2006. doi: 10.1210/endo.139.4.5933 9528987

[153] Fabbro D Pellizzari L Mercuri F Tell G Damante G. Pax-8 protein levels regulate thyroglobulin gene expression. J Mol Endocrinol (1998) 21(3):347–54. doi: 10.1677/jme.0.0210347 9845675

[154] Ortiz L Zannini M Di Lauro R Santisteban P. Transcriptional control of the forkhead thyroid transcription factor TTF-2 by thyrotropin, insulin, and insulin-like growth factor I. J Biol Chem (1997) 272(37):23334–9. doi: 10.1074/jbc.272.37.23334 9287345

[155] Saito T Endo T Nakazato M Kogai T Onaya T. Thyroid-stimulating hormone-induced down-regulation of thyroid transcription factor 1 in rat thyroid FRTL-5 cells. Endocrinology (1997) 138(2):602–6. doi: 10.1210/endo.138.2.4918 9002992

[156] Medina DL Suzuki K Pietrarelli M Okajima F Kohn LD Santisteban P. Role of insulin and serum on thyrotropin regulation of thyroid transcription factor-1 and pax-8 genes expression in FRTL-5 thyroid cells. Thyroid (2000) 10(4):295–303. doi: 10.1089/thy.2000.10.295 10807057

[157] Huang H Shi Y Liang B Cai H Cai Q. Iodinated TG in thyroid follicular lumen regulates TTF-1 and PAX8 expression *via* TSH/TSHR signaling pathway. J Cell Biochem (2017) 118(10):3444–51. doi: 10.1002/jcb.26001 28322461

[158] Kambe F Seo H. Thyroid-specific transcription factors. Endocr J (1997) 44(6):775–84. doi: 10.1507/endocrj.44.775 9622292

[159] Pellizzari L D’Elia A Rustighi A Manfioletti G Tell G Damante G. Expression and function of the homeodomain-containing protein hex in thyroid cells. Nucleic Acids Res (2000) 28(13):2503–11. doi: 10.1093/nar/28.13.2503 PMC102703 10871399

[160] Endo T Kaneshige M Nakazato M Ohmori M Harii N Onaya T. Thyroid transcription factor-1 activates the promoter activity of rat thyroid Na+/I- symporter gene. Mol Endocrinol (1997) 11(11):1747–55. doi: 10.1210/mend.11.11.0012 9328356

[161] Fernández LP López-Márquez A Martínez AM Gómez-López G Santisteban P. New insights into FoxE1 functions: identification of direct FoxE1 targets in thyroid cells. PloS One (2013) 8(5):e62849. doi: 10.1371/journal.pone.0062849 23675434 PMC3652843

[162] Ohno M Zannini M Levy O Carrasco N di Lauro R. The paired-domain transcription factor Pax8 binds to the upstream enhancer of the rat sodium/iodide symporter gene and participates in both thyroid-specific and cyclic-AMP-dependent transcription. Mol Cell Biol (1999) 19(3):2051–60. doi: 10.1128/MCB.19.3.2051 PMC83998 10022892

[163] Jang D Marcus-Samuels B Morgan SJ Klubo-Gwiezdzinska J Neumann S Gershengorn MC. Thyrotropin regulation of differentiated gene transcription in adult human thyrocytes in primary culture. Mol Cell Endocrinol (2020) 518:111032. doi: 10.1016/j.mce.2020.111032 32941925 PMC7606794

[164] Morgan SJ Neumann S Marcus-Samuels B Gershengorn MC. Thyrotropin and insulin-like growth factor 1 receptor crosstalk upregulates sodium-iodide symporter expression in primary cultures of human thyrocytes. Thyroid (2016) 26(12):1794–803. doi: 10.1089/thy.2016.0323 PMC5175432 27638195

[165] Riedel C Levy O Carrasco N. Post-transcriptional regulation of the sodium/iodide symporter by thyrotropin. J Biol Chem (2001) 276(24):21458–63. doi: 10.1074/jbc.M100561200 11290744

[166] Espinoza CR Schmitt TL Loos U. Thyroid transcription factor 1 and Pax8 synergistically activate the promoter of the human thyroglobulin gene. J Mol Endocrinol (2001) 27(1):59–67. doi: 10.1677/jme.0.0270059 11463576

[167] Zannini M Avantaggiato V Biffali E Arnone MI Sato K Pischetola M. TTF-2, a new forkhead protein, shows a temporal expression in the developing thyroid which is consistent with a role in controlling the onset of differentiation. EMBO J (1997) 16(11):3185–97. doi: 10.1093/emboj/16.11.3185 PMC1169936 9214635

[168] Perrone L Pasca di Magliano M Zannini M Di Lauro R. The thyroid transcription factor 2 (TTF-2) is a promoter-specific DNA-binding independent transcriptional repressor. Biochem Biophys Res Commun (2000) 275(1):203–8. doi: 10.1006/bbrc.2000.3232 10944465

[169] Zannini M Francis-Lang H Plachov D Di Lauro R. Pax-8, a paired domain-containing protein, binds to a sequence overlapping the recognition site of a homeodomain and activates transcription from two thyroid-specific promoters. Mol Cell Biol (1992) 12(9):4230–41. doi: 10.1128/mcb.12.9.4230-4241.1992 PMC360331 1508216

[170] Aza-Blanc P Di Lauro R Santisteban P. Identification of a cis-regulatory element and a thyroid-specific nuclear factor mediating the hormonal regulation of rat thyroid peroxidase promoter activity. Mol Endocrinol (1993) 7(10):1297–306. doi: 10.1210/mend.7.10.8264661 8264661

[171] Dentice M Luongo C Elefante A Ambrosio R Salzano S Zannini M. Pendrin is a novel *in vivo* downstream target gene of the TTF-1/Nkx-2.1 homeodomain transcription factor in differentiated thyroid cells. Mol Cell Biol (2005) 25(22):10171–82. doi: 10.1128/MCB.25.22.10171-10182.2005 PMC1280265 16260629

[172] Adler L Efrati E Zelikovic I. Molecular mechanisms of epithelial cell-specific expression and regulation of the human anion exchanger (pendrin) gene. Am J Physiol Cell Physiol (2008) 294(5):C1261–1276. doi: 10.1152/ajpcell.00486.2007 18322141

[173] Azroyan A Morla L Crambert G Laghmani K Ramakrishnan S Edwards A. Regulation of pendrin by cAMP: possible involvement in β-adrenergic-dependent NaCl retention. Am J Physiol Renal Physiol (2012) 302(9):F1180–1187. doi: 10.1152/ajprenal.00403.2011 22262479

[174] Pesce L Bizhanova A Caraballo JC Westphal W Butti ML Comellas A. TSH regulates pendrin membrane abundance and enhances iodide efflux in thyroid cells. Endocrinology (2012) 153(1):512–21. doi: 10.1210/en.2011-1548 PMC3249672 22109890

[175] Christophe-Hobertus C Christophe D. Human thyroid oxidases genes promoter activity in thyrocytes does not appear to be functionally dependent on thyroid transcription factor-1 or Pax8. Mol Cell Endocrinol (2007) 264(1-2):157–63. doi: 10.1016/j.mce.2006.11.005 17182173

[176] Bartha T Kim SW Salvatore D Gereben B Tu HM Harney JW. Characterization of the 5’-flanking and 5’-untranslated regions of the cyclic adenosine 3’,5’-monophosphate-responsive human type 2 iodothyronine deiodinase gene. Endocrinology (2000) 141(1):229–37. doi: 10.1210/endo.141.1.7282 10614643

[177] Gereben B Salvatore D Harney JW Tu HM Larsen PR. The human, but not rat, dio2 gene is stimulated by thyroid transcription factor-1 (TTF-1). Mol Endocrinol (2001) 15(1):112–24. doi: 10.1210/mend.15.1.0579 11145743

[178] Ruiz-Llorente S Carrillo Santa de Pau E Sastre-Perona A Montero-Conde C Gómez-López G Fagin JA. Genome-wide analysis of Pax8 binding provides new insights into thyroid functions. BMC Genomics (2012) 13:147. doi: 10.1186/1471-2164-13-147 22531031 PMC3403905

[179] Ambroziak M Pachucki J Stachlewska-Nasfeter E Nauman J Nauman A. Disturbed expression of type 1 and type 2 iodothyronine deiodinase as well as titf1/nkx2-1 and pax-8 transcription factor genes in papillary thyroid cancer. Thyroid (2005) 15(10):1137–46. doi: 10.1089/thy.2005.15.1137 16279847

[180] Berberich J Dietrich JW Hoermann R Müller MA. Mathematical modeling of the pituitary-thyroid feedback loop: Role of a TSH-T(3)-Shunt and sensitivity analysis. Front Endocrinol (Lausanne) (2018) 9:91. doi: 10.3389/fendo.2018.00091 29619006 PMC5871688

[181] Citterio CE Veluswamy B Morgan SJ Galton VA Banga JP Atkins S. *De novo* triiodothyronine formation from thyrocytes activated by thyroid-stimulating hormone. J Biol Chem (2017) 292(37):15434–44. doi: 10.1074/jbc.M117.784447 PMC5602401 28743746

[182] Salvatore D Tu H Harney JW Larsen PR. Type 2 iodothyronine deiodinase is highly expressed in human thyroid. J Clin Invest (1996) 98(4):962–8. doi: 10.1172/JCI118880 PMC507511 8770868

[183] Murakami M Araki O Hosoi Y Kamiya Y Morimura T Ogiwara T. Expression and regulation of type II iodothyronine deiodinase in human thyroid gland. Endocrinology (2001) 142(7):2961–7. doi: 10.1210/endo.142.7.8280 11416017

[184] Weeke J Laurberg P. 24-h profile of serum rT3 and serum 3,3’-T2 in normal man. Acta Endocrinol (Copenh) (1980) 94(4):503–6. doi: 10.1530/acta.0.0940503 7192042

[185] Russell W Harrison RF Smith N Darzy K Shalet S Weetman AP. Free triiodothyronine has a distinct circadian rhythm that is delayed but parallels thyrotropin levels. J Clin Endocrinol Metab (2008) 93(6):2300–6. doi: 10.1210/jc.2007-2674 18364382

[186] Ikuyama S Shimura H Hoeffler JP Kohn LD. Role of the cyclic adenosine 3’,5’-monophosphate response element in efficient expression of the rat thyrotropin receptor promoter. Mol Endocrinol (1992) 6(10):1701–15. doi: 10.1210/mend.6.10.1333054 1333054

[187] Civitareale D Castelli MP Falasca P Saiardi A. Thyroid transcription factor 1 activates the promoter of the thyrotropin receptor gene. Mol Endocrinol (1993) 7(12):1589–95. doi: 10.1210/mend.7.12.8145764 8145764

[188] Akamizu T Ikuyama S Saji M Kosugi S Kozak C McBride OW. Cloning, chromosomal assignment, and regulation of the rat thyrotropin receptor: expression of the gene is regulated by thyrotropin, agents that increase cAMP levels, and thyroid autoantibodies. Proc Natl Acad Sci U.S.A. (1990) 87(15):5677–81. doi: 10.1073/pnas.87.15.5677 PMC54390 1696008

[189] Saji M Akamizu T Sanchez M Obici S Avvedimento E Gottesman ME. Regulation of thyrotropin receptor gene expression in rat FRTL-5 thyroid cells. Endocrinology (1992) 130(1):520–33. doi: 10.1210/endo.130.1.1309347 1309347

[190] Shimura H Okajima F Ikuyama S Shimura Y Kimura S Saji M. Thyroid-specific expression and cyclic adenosine 3’,5’-monophosphate autoregulation of the thyrotropin receptor gene involves thyroid transcription factor-1. Mol Endocrinol (1994) 8(8):1049–69. doi: 10.1210/mend.8.8.7997232 7997232

[191] Ma K Wang H Yu J Wei M Xiao Q. New insights on the regulation of Ca(2+) -activated chloride channel TMEM16A. J Cell Physiol (2017) 232(4):707–16. doi: 10.1002/jcp.25621 27682822

[192] Liu Y Zhang H Men H Du Y Xiao Z Zhang F. Volume-regulated cl(-) current: contributions of distinct cl(-) channels and localized Ca(2+) signals. Am J Physiol Cell Physiol (2019) 317(3):C466–c480. doi: 10.1152/ajpcell.00507.2018 31242393

[193] Le SC Jia Z Chen J Yang H. Molecular basis of PIP(2)-dependent regulation of the Ca(2+)-activated chloride channel TMEM16A. Nat Commun (2019) 10(1):3769. doi: 10.1038/s41467-019-11784-8 31434906 PMC6704070

[194] Yu K Jiang T Cui Y Tajkhorshid E Hartzell HC. A network of phosphatidylinositol 4,5-bisphosphate binding sites regulates gating of the Ca(2+)-activated cl(-) channel ANO1 (TMEM16A). Proc Natl Acad Sci U.S.A. (2019) 116(40):19952–62. doi: 10.1073/pnas.1904012116 PMC6778221 31515451

[195] Tanimura Y Kiriya M Kawashima A Mori H Luo Y Kondo T. Regulation of solute carrier family 26 member 7 (Slc26a7) by thyroid stimulating hormone in thyrocytes. Endocr J (2021) 68(6):691–9. doi: 10.1507/endocrj.EJ20-0502 33583874

[196] Fischer J Kleinau G Rutz C Zwanziger D Khajavi N Müller A. Evidence of G-protein-coupled receptor and substrate transporter heteromerization at a single molecule level. Cell Mol Life Sci (2018) 75(12):2227–39. doi: 10.1007/s00018-017-2728-1 PMC11105501 29290039

[197] Venugopalan V Al-Hashimi A Weber J Rehders M Qatato M Wirth EK. The amino acid transporter Mct10/Tat1 is important to maintain the TSH receptor at its canonical basolateral localization and assures regular turnover of thyroid follicle cells in Male mice. Int J Mol Sci (2021) 22(11):5776. doi: 10.3390/ijms22115776 34071318 PMC8198332

[198] Jang D Morgan SJ Klubo-Gwiezdzinska J Banga JP Neumann S Gershengorn MC. Thyrotropin, but not thyroid-stimulating antibodies, induces biphasic regulation of gene expression in human thyrocytes. Thyroid (2020) 30(2):270–6. doi: 10.1089/thy.2019.0418 PMC7047096 31805824

[199] Deisenroth C Soldatow VY Ford J Stewart W Brinkman C LeCluyse EL. Development of an *In vitro* human thyroid microtissue model for chemical screening. Toxicol Sci (2020) 174(1):63–78. doi: 10.1093/toxsci/kfz238 PMC8061085 31808822

[200] Mangan S Alon U. Structure and function of the feed-forward loop network motif. Proc Natl Acad Sci (2003) 100(21):11980–5. doi: 10.1073/pnas.2133841100 PMC218699 14530388

[201] Laurent E Van Sande J Ludgate M Corvilain B Rocmans P Dumont JE. Unlike thyrotropin, thyroid-stimulating antibodies do not activate phospholipase c in human thyroid slices. J Clin Invest (1991) 87(5):1634–42. doi: 10.1172/JCI115178 PMC295251 1673689

[202] Boutin A Neumann S Gershengorn MC. TSH elicits cell-autonomous, biphasic responses: A mechanism inhibiting hyperstimulation. Endocrinology (2020) 161(8):1–2. doi: 10.1210/endocr/bqaa103 PMC7375800 32692808

[203] Karin O Alon U. Biphasic response as a mechanism against mutant takeover in tissue homeostasis circuits. Mol Syst Biol (2017) 13(6):933. doi: 10.15252/msb.20177599 28652282 PMC5488663

[204] Wolff J Chaikoff IL. Plasma inorganic iodide as a homeostatic regulator of thyroid function. J Biol Chem (1948) 174(2):555–64. doi: 10.1016/S0021-9258(18)57335-X 18865621

[205] Raben MS. The paradoxical effects of thiocyanate and of thyrotropin on the organic binding of iodine by the thyroid in the presence of large amounts of iodide. Endocrinology (1949) 45(3):296–304. doi: 10.1210/endo-45-3-296 18140410

[206] Stanley MM. The direct estimation of the rate of thyroid hormone formation in man. The effect of the iodide ion on thyroid iodine utilization. J Clin Endocrinol Metab (1949) 9(10):941–54. 10.1210/jcem-9-10-941 18142428

[207] Wolff J Chaikoff IL Goldberg RC Meier JR. The temporary nature of the inhibitory action of excess iodine on organic iodine synthesis in the normal thyroid. Endocrinology (1949) 45(5):504-13. doi: 10.1210/endo-45-5-504 15396709

[208] Braverman LE Ingbar SH. Changes in thyroidal function during adaptation to large doses of iodide. J Clin Invest (1963) 42(8):1216–31. doi: 10.1172/JCI104807 PMC289393 14057854

[209] Mercer CJ Sharard A Westerink CJM Adams DD. Slowing of thyroid secretion by iodide: in euthyroid people. Lancet (1960) 276(7140):19–21. doi: 10.1016/S0140-6736(60)92663-5

[210] Vagenakis AG Downs P Braverman LE Burger A Ingbar SH. Control of thyroid hormone secretion in normal subjects receiving iodides. J Clin Invest (1973) 52(2):528–32. doi: 10.1172/JCI107212 PMC302285 4683889

[211] Namba H Yamashita S Kimura H Yokoyama N Usa T Otsuru A. Evidence of thyroid volume increase in normal subjects receiving excess iodide. J Clin Endocrinol Metab (1993) 76(3):605–8. doi: 10.1210/jcem.76.3.8445017 8445017

[212] Plummer HS. Results of administering iodin to patients having exophthalmic goiter. J Am Med Assoc (1923) 80(26):1953–6.

[213] Loriaux LD. Henry S. plummer, (1874–1936). In: A biographical history of endocrinology (2016) (Hoboken, NJ: Wiley-Blackwell). p. 251–5.

[214] Phillppou G Koutras DA Plperlngos G Souvatzoglou A Moulopoulos SD. The effect of iodide on serum thyroid hormone levels in normal persons, in hyperthyroid patients, and in hypothyroid patients on thyroxine replacement. Clin Endocrinol (1992) 36(6):573–8. doi: 10.1111/j.1365-2265.1992.tb02267.x 1424182

[215] Lebsir D Manens L Grison S Lestaevel P Ebrahimian T Suhard D. Effects of repeated potassium iodide administration on genes involved in synthesis and secretion of thyroid hormone in adult male rat. Mol Cell Endocrinol (2018) 474:119–26. doi: 10.1016/j.mce.2018.02.017 29496566

[216] Eng PH Cardona GR Fang SL Previti M Alex S Carrasco N. Escape from the acute Wolff-chaikoff effect is associated with a decrease in thyroid sodium/iodide symporter messenger ribonucleic acid and protein. Endocrinology (1999) 140(8):3404–10. doi: 10.1210/endo.140.8.6893 10433193

[217] Saller B Fink H Mann K. Kinetics of acute and chronic iodine excess. Exp Clin Endocrinol Diabetes (1998) 106 Suppl 3:S34–38. doi: 10.1055/s-0029-1212044 9865552

[218] Hansson M Filipsson Nyström H Jansson S Lausmaa J Berg G. Iodine content and distribution in thyroid specimens from two patients with graves’ disease pretreated with either propylthiouracil or stable iodine: Analysis using X-ray fluorescence and time-of-Flight secondary ion mass spectrometry. Case Rep Endocrinol (2012) 2012:842357. doi: 10.1155/2012/842357 22953073 PMC3420651

[219] Bürgi H. Iodine excess. Best Pract Res Clin Endocrinol Metab (2010) 24(1):107–15. doi: 10.1016/j.beem.2009.08.010 20172475

[220] Bürgi H Radvila A Kohler H Studer H. Effects of pharmacological doses of iodide on the hyperplastic rat thyroid gland. roles of intrathyroidal iodide, thyrotropin and thyroglobulin in the Wolff-chaikoff phenomenon. Endocrinology (1974) 95(2):388–96. doi: 10.1210/endo-95-2-388 4851767

[221] Corvilain B Van Sande J Dumont JE. Inhibition by iodide of iodide binding to proteins: the “Wolff-chaikoff” effect is caused by inhibition of H2O2 generation. Biochem Biophys Res Commun (1988) 154(3):1287–92. doi: 10.1016/0006-291X(88)90279-3 2841932

[222] Panneels V Van den Bergen H Jacoby C Braekman JC Van Sande J Dumont JE. Inhibition of H2O2 production by iodoaldehydes in cultured dog thyroid cells. Mol Cell Endocrinol (1994) 102(1-2):167–76. doi: 10.1016/0303-7207(94)90110-4 7926269

[223] Juvenal J Thomasz GL Oglio R Perona M Pisarev MA Rossich L. Thyroid: Iodine beyond the thyronines. Curr Chem Biol (2011) 5(3):163–7. doi: 10.2174/2212796811105030163

[224] Ohayon R Boeynaems JM Braekman JC Van den Bergen H Gorin Y Virion A. Inhibition of thyroid NADPH-oxidase by 2-iodohexadecanal in a cell-free system. Mol Cell Endocrinol (1994) 99(1):133–41. doi: 10.1016/0303-7207(94)90156-2 8187956

[225] Rossich LE Thomasz L Nicola JP Nazar M Salvarredi LA Pisarev M. Effects of 2-iodohexadecanal in the physiology of thyroid cells. Mol Cell Endocrinol (2016) 437:292–301. doi: 10.1016/j.mce.2016.08.036 27568464

[226] Koukkou EG Roupas ND Markou KB. Effect of excess iodine intake on thyroid on human health. Minerva Med (2017) 108(2):136–46. doi: 10.23736/S0026-4806.17.04923-0 28079354

[227] Yoshinari M Inoue K Nakashima T Okamura K Shiroozu A Nishitani H. Acid protease activity in thyroid gland from patients with graves’ disease. Metabolism (1983) 32(4):348–54. doi: 10.1016/0026-0495(83)90042-2 6353140

[228] Lamas L Ingbar SH. The effect of varying iodine content on the susceptibility of thyroglobulin to hydrolysis by thyroid acid protease. Endocrinology (1978) 102(1):188–97. doi: 10.1210/endo-102-1-188 33793

[229] Starling JR Hopps BA. Effect of excess iodine on thyroid and liver lysosomal enzymes. J Surg Res (1980) 28(1):57–64. doi: 10.1016/0022-4804(80)90083-9 7359909

[230] Sinadinović J Kraincanić M Marinković B Jovanović M Petrović N Liewendahl K. Susceptibility to proteolysis of thyroglobulin from rats and guinea-pigs treated with excess iodide. Acta Endocrinol (Copenh) (1982) 99(2):232–8. doi: 10.1530/acta.0.0990232 7036631

[231] Sinadinović J Liewendahl K. Studies on proteolytic activity and function of the thyroid gland in rats administered excess iodide. Acta Endocrinol (Copenh) (1976) 82(4):728–36. doi: 10.1530/acta.0.0820728 947282

[232] Ahad F Ganie SA. Iodine, iodine metabolism and iodine deficiency disorders revisited. Indian J Endocrinol Metab (2010) 14(1):13–7. PMC3063534 21448409

[233] Becker DV. Reactor accidents. public health strategies and their medical implications. Jama (1987) 258(5):649–54. doi: 10.1001/jama.1987.03400050091033 3612987

[234] Eng PH Cardona GR Previti MC Chin WW Braverman LE. Regulation of the sodium iodide symporter by iodide in FRTL-5 cells. Eur J Endocrinol (2001) 144(2):139–44. doi: 10.1530/eje.0.1440139 11182750

[235] Leoni SG Galante PA Ricarte-Filho JC Kimura ET. Differential gene expression analysis of iodide-treated rat thyroid follicular cell line PCCl3. Genomics (2008) 91(4):356–66. doi: 10.1016/j.ygeno.2007.12.009 18272324

[236] Suzuki K Kimura H Wu H Kudo N Kim WB Suzuki S. Excess iodide decreases transcription of NIS and VEGF genes in rat FRTL-5 thyroid cells. Biochem Biophys Res Commun (2010) 393(2):286–90. doi: 10.1016/j.bbrc.2010.01.123 PMC2834827 20132794

[237] Serrano-Nascimento C Nicola JP Teixeira Sda S Poyares LL Lellis-Santos C Bordin S. Excess iodide downregulates Na(+)/I(-) symporter gene transcription through activation of PI3K/Akt pathway. Mol Cell Endocrinol (2016) 426:73–90. doi: 10.1016/j.mce.2016.02.006 26872612

[238] Serrano-Nascimento C da Silva Teixeira S Nicola JP Nachbar RT Masini-Repiso AM Nunes MT. The acute inhibitory effect of iodide excess on sodium/iodide symporter expression and activity involves the PI3K/Akt signaling pathway. Endocrinology (2014) 155(3):1145–56. doi: 10.1210/en.2013-1665 24424051

[239] Serrano-Nascimento C Calil-Silveira J Nunes MT. Posttranscriptional regulation of sodium-iodide symporter mRNA expression in the rat thyroid gland by acute iodide administration. Am J Physiol Cell Physiol (2010) 298(4):C893–899. doi: 10.1152/ajpcell.00224.2009 20107044

[240] Munroe D Jacobson A. Tales of poly(A): a review. Gene (1990) 91(2):151–8. doi: 10.1016/0378-1119(90)90082-3 1976572

[241] Grollman EF Smolar A Ommaya A Tombaccini D Santisteban P. Iodine suppression of iodide uptake in FRTL-5 thyroid cells. Endocrinology (1986) 118(6):2477–82. doi: 10.1210/endo-118-6-2477 3009160

[242] Ferreira AC Lima LP Araújo RL Müller G Rocha RP Rosenthal D. Rapid regulation of thyroid sodium-iodide symporter activity by thyrotrophin and iodine. J Endocrinol (2005) 184(1):69–76. doi: 10.1677/joe.1.05643 15642784

[243] Calil-Silveira J Serrano-Nascimento C Nunes MT. Iodide treatment acutely increases pendrin (SLC26A4) mRNA expression in the rat thyroid and the PCCl3 thyroid cell line by transcriptional mechanisms. Mol Cell Endocrinol (2012) 350(1):118–24. doi: 10.1016/j.mce.2011.12.002 22178794

[244] Calil-Silveira J Serrano-Nascimento C Kopp PA Nunes MT. Iodide excess regulates its own efflux: a possible involvement of pendrin. Am J Physiol Cell Physiol (2016) 310(7):C576–582. doi: 10.1152/ajpcell.00210.2015 PMC4971809 26791486

[245] Wang K Sun YN Liu JY Zhang L Ye Y Lin LX. The impact of iodine excess on thyroid hormone biosynthesis and metabolism in rats. Biol Trace Elem. Res (2009) 130(1):72–85. doi: 10.1007/s12011-009-8315-z 19214402

[246] Solis SJ Villalobos P Orozco A Delgado G Quintanar-Stephano A Garcia-Solis P. Inhibition of intrathyroidal dehalogenation by iodide. J Endocrinol (2011) 208(1):89–96. doi: 10.1677/JOE-10-0300 20974636

[247] Suzuki K Mori A Lavaroni S Ulianich L Miyagi E Saito J. Thyroglobulin regulates follicular function and heterogeneity by suppressing thyroid-specific gene expression. Biochimie (1999) 81(4):329–40. doi: 10.1016/S0300-9084(99)80078-9 10401666

[248] Suzuki K Mori A Lavaroni S Miyagi E Ulianich L Katoh R. *In vivo* expression of thyroid transcription factor-1 RNA and its relation to thyroid function and follicular heterogeneity: identification of follicular thyroglobulin as a feedback suppressor of thyroid transcription factor-1 RNA levels and thyroglobulin synthesis. Thyroid (1999) 9(4):319–31. doi: 10.1089/thy.1999.9.319 10319936

[249] Suzuki K Kohn LD. Differential regulation of apical and basal iodide transporters in the thyroid by thyroglobulin. J Endocrinol (2006) 189(2):247–55. doi: 10.1677/joe.1.06677 16648292

[250] Yoshihara A Hara T Kawashima A Akama T Tanigawa K Wu H. Regulation of dual oxidase expression and H2O2 production by thyroglobulin. Thyroid (2012) 22(10):1054–62. doi: 10.1089/thy.2012.0003 PMC3462396 22874065

[251] Ishido Y Yamazaki K Kammori M Sugishita Y Luo Y Yamada E. Thyroglobulin suppresses thyroid-specific gene expression in cultures of normal but not neoplastic human thyroid follicular cells. J Clin Endocrinol Metab (2014) 99(4):E694–702. doi: 10.1210/jc.2013-3682 24433000

[252] Suzuki K Mori A Saito J Moriyama E Ullianich L Kohn LD. Follicular thyroglobulin suppresses iodide uptake by suppressing expression of the sodium/iodide symporter gene. Endocrinology (1999) 140(11):5422–30. doi: 10.1210/endo.140.11.7124 10537174

[253] Kohn LD Suzuki K Nakazato M Royaux I Green ED. Effects of thyroglobulin and pendrin on iodide flux through the thyrocyte. Trends Endocrinol Metab (2001) 12(1):10–6. doi: 10.1016/S1043-2760(00)00337-4 11137035

[254] Royaux IE Suzuki K Mori A Katoh R Everett LA Kohn LD. Pendrin, the protein encoded by the pendred syndrome gene (PDS), is an apical porter of iodide in the thyroid and is regulated by thyroglobulin in FRTL-5 cells. Endocrinology (2000) 141(2):839–45. doi: 10.1210/endo.141.2.7303 10650967

[255] Oda K Luo Y Yoshihara A Ishido Y Sekihata K Usukura K. Follicular thyroglobulin induces cathepsin h expression and activity in thyrocytes. Biochem Biophys Res Commun (2017) 483(1):541–6. doi: 10.1016/j.bbrc.2016.12.109 27998776

[256] Ishido Y Luo Y Yoshihara A Hayashi M Yoshida A Hisatome I. Follicular thyroglobulin enhances gene expression necessary for thyroid hormone secretion. Endocr J (2015) 62(11):1007–15. doi: 10.1507/endocrj.EJ15-0263 26370556

[257] Sellitti DF Suzuki K. Intrinsic regulation of thyroid function by thyroglobulin. Thyroid (2014) 24(4):625–38. doi: 10.1089/thy.2013.0344 PMC3993028 24251883

[258] Huang H Shi Y Lin L Li L Lin X Li X. Inhibition of thyroid-restricted genes by follicular thyroglobulin involves iodinated degree. J Cell Biochem (2011) 112(3):971–7. doi: 10.1002/jcb.23014 21308730

[259] Nakazato M Chung HK Ulianich L Grassadonia A Suzuki K Kohn LD. Thyroglobulin repression of thyroid transcription factor 1 (TTF-1) gene expression is mediated by decreased DNA binding of nuclear factor I proteins which control constitutive TTF-1 expression. Mol Cell Biol (2000) 20(22):8499–512. doi: 10.1128/MCB.20.22.8499-8512.2000 PMC102156 11046146

[260] Suzuki K Kawashima A Yoshihara A Akama T Sue M Yoshida A. Role of thyroglobulin on negative feedback autoregulation of thyroid follicular function and growth. J Endocrinol (2011) 209(2):169–74. doi: 10.1530/JOE-10-0486 21378092

[261] Studer H Peter HJ Gerber H. Natural heterogeneity of thyroid cells: the basis for understanding thyroid function and nodular goiter growth. Endocr Rev (1989) 10(2):125–35. doi: 10.1210/edrv-10-2-125 2666115

[262] Aeschimann S Kopp PA Kimura ET Zbaeren J Tobler A Fey MF. Morphological and functional polymorphism within clonal thyroid nodules. J Clin Endocrinol Metab (1993) 77(3):846–51. doi: 10.1210/jcem.77.3.8370709 8370709

[263] Studer H Forster R Conti A Kohler H Haeberli A Engler H. Transformation of normal follicles into thyrotropin-refractory “cold” follicles in the aging mouse thyroid gland. Endocrinology (1978) 102(5):1576–86. doi: 10.1210/endo-102-5-1576 744040

[264] Gerber H Peter HJ Studer H. Age-related failure of endocytosis may be the pathogenetic mechanism responsible for “cold” follicle formation in the aging mouse thyroid. Endocrinology (1987) 120(5):1758–64. doi: 10.1210/endo-120-5-1758 3569110

[265] Gerber H Studer H von Grünigen C. Paradoxical effects of thyrotropin on diffusion of thyroglobulin in the colloid of rat thyroid follicles after long term thyroxine treatment. Endocrinology (1985) 116(1):303–10. doi: 10.1210/endo-116-1-303 3964748

[266] Peter HJ Gerber H Studer H Smeds S. Pathogenesis of heterogeneity in human multinodular goiter. a study on growth and function of thyroid tissue transplanted onto nude mice. J Clin Invest (1985) 76(5):1992–2002. doi: 10.1172/JCI112199 PMC424262 4056062

[267] Gerber H Peter HJ Studer H. Diffusion of thyroglobulin in the follicular colloid. (Minireview). Endocrinol Exp (1986) 20(1):23–33. 3516644

[268] Roger PP Baptist M Dumont JE. A mechanism generating heterogeneity in thyroid epithelial cells: suppression of the thyrotropin/cAMP-dependent mitogenic pathway after cell division induced by cAMP-independent factors. J Cell Biol (1992) 117(2):383–93. doi: 10.1083/jcb.117.2.383 PMC2289413 1313816

[269] Huang H Chen L Liang B Cai H Cai Q Shi Y. Upregulation of TSHR, TTF-1, and PAX8 in nodular goiter is associated with iodine deficiency in the follicular lumen. Int J Endocrinol (2016) 2016:2492450. doi: 10.1155/2016/2492450 27525008 PMC4976194

[270] Chen F Wang H Li Q Li Z Luo Y. Progress in the research of negative feedback effect of thyroglobulin. Nan Fang Yi Ke Da Xue Xue Bao (2019) 39(1):125–6. doi: 10.12122/j.issn.1673-4254.2019.01.20 PMC6765574 30692078

[271] Novák B Tyson JJ. Design principles of biochemical oscillators. Nat Rev Mol Cell Biol (2008) 9(12):981–91. doi: 10.1038/nrm2530 PMC2796343 18971947

[272] Ferrell JE Jr. Self-perpetuating states in signal transduction: positive feedback, double-negative feedback and bistability. Curr Opin Cell Biol (2002) 14(2):140–8. doi: 10.1016/S0955-0674(02)00314-9 11891111

[273] Saratchandran P Carson ER Reeve J. An improved mathematical model of human thyroid hormone regulation. Clin Endocrinol (Oxf) (1976) 5(5):473–83. doi: 10.1111/j.1365-2265.1976.tb01976.x 825330

[274] Liu Y Liu B Xie J Liu YX. A new mathematical model of hypothalamo-pituitary-thyroid axis. Math Comput Model (1994) 19(9):81–90. doi: 10.1016/0895-7177(94)90042-6

[275] Eisenberg M Samuels M DiStefano JJ 3rd. Extensions, validation, and clinical applications of a feedback control system simulator of the hypothalamo-pituitary-thyroid axis. Thyroid (2008) 18(10):1071–85. doi: 10.1089/thy.2007.0388 PMC2962855 18844475

[276] Degon M Chipkin SR Hollot CV Zoeller RT Chait Y. A computational model of the human thyroid. Math Biosci (2008) 212(1):22–53. doi: 10.1016/j.mbs.2007.10.009 18291425

[277] Cohen DPA Lebsir D Benderitter M Souidi M. A systems biology approach to propose a new mechanism of regulation of repetitive prophylaxis of stable iodide on sodium/iodide symporter (NIS). Biochimie (2019) 162:208–15. doi: 10.1016/j.biochi.2019.04.024 31071356

[278] Leonard JA Tan YM Gilbert M Isaacs K El-Masri H. Estimating margin of exposure to thyroid peroxidase inhibitors using high-throughput *in vitro* data, high-throughput exposure modeling, and physiologically based Pharmacokinetic/Pharmacodynamic modeling. Toxicol Sci (2016) 151(1):57–70. doi: 10.1093/toxsci/kfw022 PMC4914794 26865668

[279] Conolly RB Ankley GT Cheng W Mayo ML Miller DH Perkins EJ. Quantitative adverse outcome pathways and their application to predictive toxicology. Environ Sci Technol (2017) 51(8):4661–72. doi: 10.1021/acs.est.6b06230 PMC6134852 28355063

[280] Handa S Hassan I Gilbert M El-Masri H. Mechanistic computational model for extrapolating *in vitro* thyroid peroxidase (TPO) inhibition data to predict serum thyroid hormone levels in rats. Toxicol Sci (2021) 183(1):36–48. doi: 10.1093/toxsci/kfab074 PMC8771814 34117770

[281] Svingen T Villeneuve DL Knapen D Panagiotou EM Draskau MK Damdimopoulou P. A pragmatic approach to adverse outcome pathway development and evaluation. Toxicol. Sci (2021) 184(2):183–90. doi: 10.1093/toxsci/kfab113 PMC8633887 34534351

